# Storm impacts on phytoplankton community dynamics in lakes

**DOI:** 10.1111/gcb.15033

**Published:** 2020-03-05

**Authors:** Jason D. Stockwell, Jonathan P. Doubek, Rita Adrian, Orlane Anneville, Cayelan C. Carey, Laurence Carvalho, Lisette N. De Senerpont Domis, Gaël Dur, Marieke A. Frassl, Hans‐Peter Grossart, Bas W. Ibelings, Marc J. Lajeunesse, Aleksandra M. Lewandowska, María E. Llames, Shin‐Ichiro S. Matsuzaki, Emily R. Nodine, Peeter Nõges, Vijay P. Patil, Francesco Pomati, Karsten Rinke, Lars G. Rudstam, James A. Rusak, Nico Salmaso, Christian T. Seltmann, Dietmar Straile, Stephen J. Thackeray, Wim Thiery, Pablo Urrutia‐Cordero, Patrick Venail, Piet Verburg, R. Iestyn Woolway, Tamar Zohary, Mikkel R. Andersen, Ruchi Bhattacharya, Josef Hejzlar, Nasime Janatian, Alfred T. N. K. Kpodonu, Tanner J. Williamson, Harriet L. Wilson

**Affiliations:** ^1^ Rubenstein Ecosystem Science Laboratory University of Vermont Burlington VT USA; ^2^ Department of Ecosystem Research Leibniz Institute of Freshwater Ecology and Inland Fisheries Berlin Germany; ^3^ Department of Biology, Chemistry and Pharmacy Freie Universität Berlin Berlin Germany; ^4^ CARRTEL INRAE University Savoie Mont Blanc Thonon‐les‐Bains France; ^5^ Biological Sciences Virginia Tech Blacksburg VA USA; ^6^ Freshwater Restoration & Sustainability Group UK Centre for Ecology & Hydrology Penicuik Midlothian UK; ^7^ Aquatic Ecology Netherlands Institute of Ecology Wageningen The Netherlands; ^8^ Creative Science Unit (Geoscience) Faculty of Science Shizuoka University Surugaku Japan; ^9^ Australian Rivers Institute Griffith University Nathan Qld Australia; ^10^ Department of Experimental Limnology Leibniz Institute for Freshwater Ecology and Inland Fisheries Stechlin Germany; ^11^ Institute of Biochemistry and Biology Potsdam University Potsdam Germany; ^12^ Department F.‐A. Forel for Environmental and Aquatic Sciences Institute for Environmental Sciences University of Geneva Geneva Switzerland; ^13^ Department of Integrative Biology University of South Florida Tampa FL USA; ^14^ Tvärminne Zoological Station University of Helsinki Hanko Finland; ^15^ Laboratorio de Ecología Acuática Instituto Tecnológico Chascomús (INTECH) (UNSAM‐CONICET) Chascomús Buenos Aires Argentina; ^16^ Center for Environmental Biology & Ecosystem Studies National Institute for Environmental Studies Tsukuba Japan; ^17^ Environmental Studies Rollins College Winter Park FL USA; ^18^ Institute of Agricultural and Environmental Sciences Estonian University of Life Sciences Tartu Estonia; ^19^ Alaska Science Center US Geological Survey Anchorage AK USA; ^20^ Aquatic Ecology Eawag, Swiss Federal Institute of Water Science and Technology Dubendorf Switzerland; ^21^ Lake Research Helmholtz‐Centre for Environmental Research Magdeburg Germany; ^22^ Department of Natural Resources Cornell University Ithaca NY USA; ^23^ Dorset Environmental Science Centre Ontario Ministry of Environment, Conservation and Parks Dorset ON Canada; ^24^ Department of Biology Queen's University Kingston ON Canada; ^25^ Department of Sustainable Agro‐ecosystems and Bioresources, Research and Innovation Centre Fondazione Edmund Mach San Michele all'Adige Italy; ^26^ Department of Biology Limnological Institute University of Konstanz Konstanz Germany; ^27^ Lake Ecosystems Group UK Centre for Ecology & Hydrology Lancaster Environment Centre Lancaster UK; ^28^ Institute for Atmospheric and Climate Science ETH Zürich Zürich Switzerland; ^29^ Department of Hydrology and Hydraulic Engineering Vrije Universiteit Brussel Brussels Belgium; ^30^ Department of Ecology and Genetics Uppsala University Uppsala Sweden; ^31^ Helmholtz Institute for Functional Marine Biodiversity (HIFMB) Oldenburg Germany; ^32^ Institute for Chemistry and Biology of Marine Environments (ICBM) Carl‐von‐Ossietzky University Oldenburg Wilhelmshaven Germany; ^33^ National Institute of Water and Atmospheric Research Hamilton New Zealand; ^34^ Centre for Freshwater and Environmental Studies Dundalk Institute of Technology Dundalk Ireland; ^35^ Kinneret Limnological Laboratory Israel Oceanographic & Limnological Research Migdal Israel; ^36^ The Water Institute University of Waterloo Waterloo ON Canada; ^37^ Institute of Hydrobiology Biology Centre of the Czech Academy of Sciences České Budějovice Czechia; ^38^ Research Foundation City University of New York New York NY USA; ^39^ Department of Biology Miami University Oxford OH USA; ^40^ Present address: School of Natural Resources & Environment Lake Superior State University Sault Sainte Marie MI 49783 USA; ^41^ Present address: Center for Freshwater Research and Education Lake Superior State University Sault Sainte Marie MI 49783 USA; ^42^ Present address: Environmental Engineering Department Universidad de Ingenieria y Tecnologia (UTEC) Lima Peru

**Keywords:** climate change, environmental disturbance, extreme events, functional traits, mixing, nutrients, stability, watershed

## Abstract

In many regions across the globe, extreme weather events such as storms have increased in frequency, intensity, and duration due to climate change. Ecological theory predicts that such extreme events should have large impacts on ecosystem structure and function. High winds and precipitation associated with storms can affect lakes via short‐term runoff events from watersheds and physical mixing of the water column. In addition, lakes connected to rivers and streams will also experience flushing due to high flow rates. Although we have a well‐developed understanding of how wind and precipitation events can alter lake physical processes and some aspects of biogeochemical cycling, our mechanistic understanding of the emergent responses of phytoplankton communities is poor. Here we provide a comprehensive synthesis that identifies how storms interact with lake and watershed attributes and their antecedent conditions to generate changes in lake physical and chemical environments. Such changes can restructure phytoplankton communities and their dynamics, as well as result in altered ecological function (e.g., carbon, nutrient and energy cycling) in the short‐ and long‐term. We summarize the current understanding of storm‐induced phytoplankton dynamics, identify knowledge gaps with a systematic review of the literature, and suggest future research directions across a gradient of lake types and environmental conditions.

## Graphical abstract

Our understanding of ecosystem‐scale responses to storm events is fragmented, and terminological variability in defining a “storm” hinders resolution of generalizable impacts. We provide a comprehensive synthesis of the interactions between physical properties and biological responses in lakes that demonstrates the context‐dependency of storm impacts; lake/watershed attributes and their antecedent conditions mediate the extent to which storms impact lake ecosystems. We develop a framework which conceptualizes how abrupt, storm‐induced changes in the lake environment influence phytoplankton community dynamics through functional traits, identifies current limitations, and highlights several avenues of research to narrow knowledge gaps in synthetic and interdisciplinary ways.

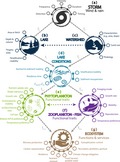

## 1 INTRODUCTION

Extreme weather events (EWEs) are ranked as the highest global risk in terms of likelihood of occurrence and third highest in impact, ranking behind only weapons of mass destruction and failure to mitigate and adapt to climate change (World Economic Forum, 2019). The transient and lasting effects of EWEs (including droughts, heat waves, and storms) on ecosystems are undeniable (Coumou & Rahmstorf, 2012; Knapp et al., 2002; Nielsen & Ball, 2015; Thibault & Brown, 2008; Zheng, Xue, Li, Chen, & Tao, 2016), but are much less understood than the effects of longer‐term changes in average environmental conditions (Carvalho et al., 2012; Jentsch, Kreyling, & Beierkuhnlein, 2007; Jeppesen et al., 2005; Parmesan, Root, & Willig, 2000; Walther et al., 2002). EWEs and changes in their frequency, intensity, and duration may be just as important as these longer‐term changes for ecological and evolutionary processes (Jentsch et al., 2007; Lawson, Vindenes, Bailey, & van de Pol, 2015; Vasseur et al., 2014), encompassing levels of organization from genes to ecosystems (Ehrlich et al., 1980; Gutschick & BassiriRad, 2003; Knapp et al., 2015). Despite a decline in average wind speeds over most continental areas in recent decades (Bichet, Wild, Folini, & Schar, 2012; McVicar & Roderick, 2010; Vautard, Cattiaux, Yiou, Thépaut, & Ciais, 2010), the frequency, intensity, and duration of storms have increased over the same period (Lehmann, Coumou, & Frieler, 2015; Webster, Holland, Curry, & Chang, 2005; Zhang, Wan, Zwiers, Hegerl, & Min, 2013) and are generally projected to continue to increase (Bacmeister et al., 2018; Coumou & Rahmstorf, 2012; Fischer & Knutti, 2015; Thiery et al., 2016). Consequently, development and understanding of mechanistic links between storms and short‐ to long‐term responses of ecosystem structure and function are critical areas of inquiry (Parmesan, 2006; Ummenhofer & Meehl, 2017; van de Pol, Jenouvrier, Cornelissen, & Visser, 2017).

Lakes can be sensitive to storm events because they integrate information across watersheds (Adrian et al., 2009; Williamson, Saros, & Schindler, 2009). Their rapid responses to pulses of storm energy (Brothers et al., 2014; de Castro Medeiros, Mattos, Lürling, & Becker, 2015; Robarts, Waiser, Hadas, Zohary, & MacIntyre, 1998; Rosenzweig et al., 2007) provide opportunities to (a) explore, test, and refine ecological concepts over readily observable time scales (Padisák, Tóth, & Rajczy, 1988, 1990); and (b) expand our perspective of climate change impacts beyond trends in average environmental conditions (Cohen et al., 2016; O'Reilly et al., 2015), to consider the role of increased environmental variability on ecosystem functioning (Jennings et al., 2012; Reynolds, 1993).

Wind and precipitation events can quickly alter light, nutrient, and temperature conditions in lakes (Kuha et al., 2016; Sadro & Melack, 2012; Tsai et al., 2008), which are the key determinants of algal growth (Conley et al., 2009; Dickman, Vanni, & Horgan, 2006; Kirk, 2010; Patrick, 1969; Schindler, 2006; Talling, 1971). Algae and cyanobacteria, the foundation of aquatic food webs, are highly diverse in their taxonomy and functional traits. Storm‐induced changes to lake abiotic conditions and physical displacement of phytoplankton throughout the water column could drive the outcome of phytoplankton species competition and thus shape community composition (Huisman et al., 2004; Reynolds, Huszar, Kruk, Naselli‐Flores, & Melo, 2002; Smith, 1983) and food web dynamics (Ceulemans, Gaedke, Klauschies, & Guill, 2019; Ellner, Geber, & Hairston, 2011; Tirok & Gaedke, 2010). Traits such as size and morphology, life history, physiological responses, and adaptive capacity (Padisák, 2004; Reynolds, 2006) mediate phytoplankton survival, competition, growth, and reproduction (Litchman, de Tezanos Pinto, Klausmeier, Thomas, & Yoshiyama, 2010). The fast growth rates and short generation times of phytoplankton enable fast responses to abrupt, storm‐induced changes in the lakes (Jacobsen & Simonsen, 1993; Padisák, Tóth, & Rajczy, 1988; Reynolds, 1984, 1988a). Rapid changes in phytoplankton community composition, diversity, and primary production (Lewis Jr., 1974; Padisák, 1993; Reynolds, 1988a, 1993) could subsequently alter ecosystem function and services (Carpenter et al., 1998; Conley et al., 2009; Schindler, 2006). Given the potential sensitivity of phytoplankton to storm‐induced perturbations, development of research on the impact of storms is increasingly urgent (Bergkemper & Weisse, 2018; Marcé et al., 2016).

Biological responses to storms may manifest at one or more levels of ecological organization (e.g., from individuals to ecosystems; Felton & Smith, 2017; Gutschick & BassiriRad, 2003; Havens et al., 2011; van de Pol et al., 2017), whereas the timescale of the response may be immediate or delayed (Foreman, Wolf, & Priscu, 2004; Giling et al., 2017; Klug et al., 2012) and may last from minutes to decades (Bachmann, Hoyer, & Canfield Jr., 1999; Lohrenz et al., 2004; Perga, Bruel, Rodriguez, Guénand, & Bouffard, 2018; Rusak et al., 2018). We argue that functional trait‐based approaches (Litchman & Klausmeier, 2008; Padisák, Crossetti, & Naselli‐Flores, 2009; Salmaso, Naselli‐Flores, & Padisák, 2015), based on life‐history concepts of r‐ and K‐strategists (Margalef, 1978) and later on competitive, stress‐tolerant, and ruderal (C‐S‐R) strategists (Reynolds, 1988b), provide a starting framework to understand and predict the temporal dynamics of ecological and evolutionary responses of phytoplankton (sensu Reznick, Losos, & Travis, 2019) to storm‐induced changes in light, nutrients, and temperature.

The role of storms in shaping phytoplankton community dynamics is context‐dependent (sensu Huston, 2014), and thus the responses of phytoplankton communities to storm disturbances are influenced by many factors, including lake typology, abiotic and biotic conditions, and extant phytoplankton community composition (including propagule banks in the sediment; Reynolds, Padisák, & Sommer, 1993). Tychoplankton may be suspended in the water column during storm events, which increase mixing via wind or precipitation (Schelske, Carrick, & Aldridge, 1995). Wind events affect shallow versus deep lakes differently, based on interactions between mixing depth and the lake bottom (Andersen, Sand‐Jensen, Woolway, & Jones, 2017; Delandmeter et al., 2018; Robarts et al., 1998), and precipitation events and subsequent runoff and flushing have different impacts on reservoirs, shallow lakes, and deep lakes based on variations in water residence times (Doubek & Carey, 2017; Elliott, 2010; Hayes, Deemer, Corman, Razavi, & Strock, 2017; Søballe & Kimmel, 1987; Wetzel, 1990) and the water layer into which the inflow penetrates (Vilhena, Hillmer, & Imberger, 2010). Consequently, the environmental and ecological contexts of lakes are required to better evaluate and predict the effects of storm disturbances on phytoplankton community dynamics.

In this paper, we present a synthesis of the impacts of storms on lakes using a two‐step rationale, considering (a) the effects of wind and precipitation on the physical and chemical structure of the water column (i.e., light, temperature, and nutrients), as mediated by lake and watershed attributes; and (b) their expected importance in shaping lake phytoplankton community dynamics, based on hierarchical taxonomic levels and functional traits including established life‐history and functional association concepts. Overall, our goal is to provide a comprehensive and mechanistic understanding of possible phytoplankton responses to storm‐induced disturbances, and to generate a testable framework that could help guide future research.

## 2 STORMS AND LIMNOLOGY

Storms are associated with heavy precipitation, strong wind, and the passage of warmer or cooler air masses (Easterling et al., 2000; Hegerl, Hanlon, & Beierkuhnlein, 2011; MacIntyre, Sickman, Goldthwait, & Kling, 2006). In strict meteorological terminology, a storm is “an atmospheric disturbance involving perturbations of the prevailing pressure and wind fields, on scales ranging from tornadoes (1 km across) to extratropical cyclones (2000–3000 km across)” and/or “wind with a speed between 48 and 55 knots (25 and 28 m/s; Beaufort scale wind force 10)” (World Meteorological Organization, 1967, p. 148). In practice, however, storm definitions are highly variable depending on the type of storm, region, and discipline, and thus often refer to different baselines, that is, average wind speed across regions (Read et al., 2011) or deviation from the average (Jennings et al., 2012). Furthermore, storms are typically framed in terms of impacts on humans, often with reference to destruction of property and human life (Beniston et al., 2007).

Most ecological research on the effects of extreme events, including storms, has been based on meteorological forcing metrics (e.g., top 5% of wind events) rather than being conceptualized in terms of ecological impacts (van de Pol et al., 2017). However, not all forcing events need to be extreme (and thus rare) to be biologically impactful and not all extreme forcing events will have a biological impact (Bailey & van de Pol, 2016; van de Pol et al., 2017). For instance, an extreme wind event may have little impact on phytoplankton in a lake which was fully mixed prior to the event, whereas storm effects on phytoplankton community assembly may compound (sensu Leonard et al., 2014) when lakes are not yet recovered from a previous storm. Hence the timing of storm events, and antecedent conditions, may greatly influence the ecological impact of storms (Perga et al., 2018).

To assess how studies have defined and used the term “storm” in relation to phytoplankton dynamics, we conducted a systematic review of the literature. After screening the titles and abstracts of the initial 4,346 papers identified through a Web of Science search (1961–2017) using the terms phytoplank* and (storm* or wind* or hurricane* or monsoon* or cyclone* or disturbance*), 309 were identified to contain potentially relevant content in terms of storm effects on phytoplankton (see Supporting Information for further details on our screening and coding protocols; also see Lajeunesse, 2016).

“Storm” was used in 118 of the 309 (38.2%) papers, but definitions were found in only 38. Some papers described storms as extreme wind‐related events, others used extreme precipitation, and others used a combination of both (Table S2). In some cases, the meteorological terms hurricane and typhoon were used. For wind, a storm was often defined using general descriptors such as strong winds or gusts of an episodic nature (e.g., greater than the seasonal average) or increases in daily mean wind speed. Other papers used wind thresholds ranging from 4 to >20 m/s to define storms. Intense precipitation was also used to define storms and studies typically provided quantities of total rainfall over a defined period ranging from as little as 6 to over 100 mm/day (Table S2). In one case, estimates of the amount of rainfall relative to the total lake volume were provided (33% and 50% of lake volume; Table S2). Therefore, the definition of a storm, when provided, was highly variable.

Only 25 of the 309 papers met our criteria of simultaneously reporting (a) storm effects on (b) physics/chemistry of lakes, reservoirs, or ponds with (c) evaluation of phytoplankton responses (see Supporting Information for details). The 25 papers reported *31 different studies* of phytoplankton responding to changes in lake physics or chemistry from storms—some papers included studies of multiple lakes (Paidere, Gruberts, Škute, & Druvietis, 2007), storm events (Znachor, Zapomēlová, Reháková, Nedoma, & Šimek, 2008), or time periods (Li, Huang, Ma, Sun, & Zhang, 2015), or distinct basins within the same lake (Robarts et al., 1998; Table 1). The 31 studies included 18 lakes and five reservoirs in 14 countries across Europe (9), East Asia (3), and North America (2). Surface areas ranged from 0.038 to 2,339 km^2^ and mean depths ranged from 1.7 to 100 m. Trophic states ranged from oligotrophic to hypereutrophic, with more than half of the water bodies reported as eutrophic or hypereutrophic (Table 1).

**FIGURE 1:**
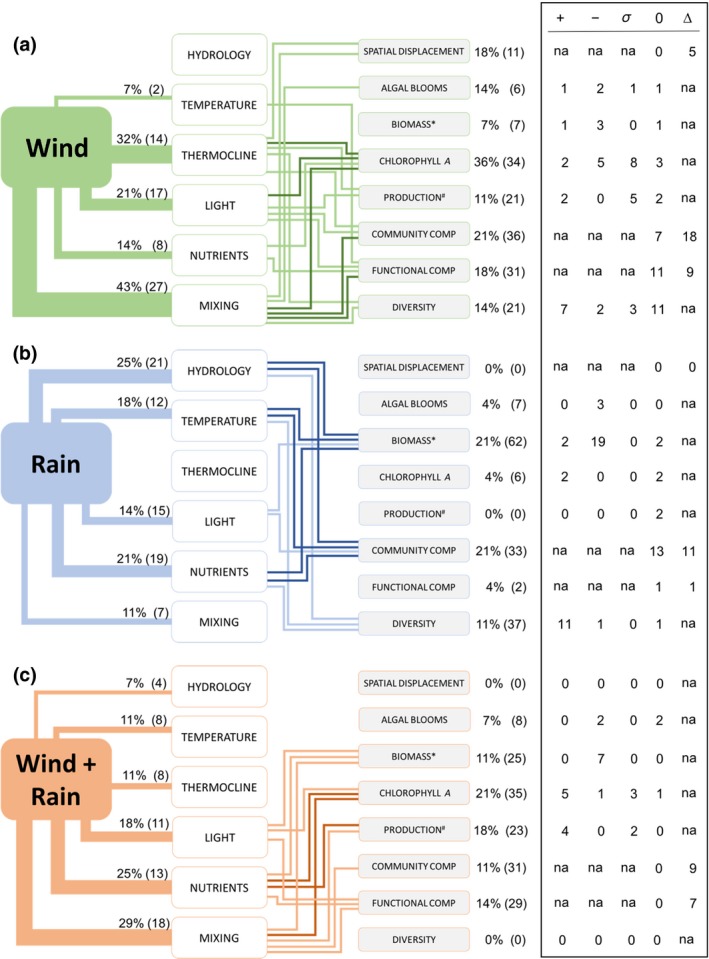
Summary of the systematic review linking three types of storm events, wind (a), rain (b), and wind plus rain (c), to six variables related to lake chemical and physical condition (center column) and their consequent links to eight phytoplankton variables (right column). For details see Table 1. The connectors between different variables represent the links described by the authors in the studies or supported by data presented in the publications. The width of the connectors between weather events and lake conditions is proportional to the percent occurrence of each link in the studies which met our criteria. The percent occurrence and the total number of reported links (in parentheses) are located above the connectors. For clarity, only connectors between the lake condition variables and the phytoplankton‐related variables that were reported in at least 9% (lighter connectors) or more than 16% (darker connectors) of the studies were included in the figure. The numbers to the right of the phytoplankton‐related variables represent the percent occurrence and total number of links (in parentheses) in which each phytoplankton‐related variable was found. The table to the right indicates the number of storm events which resulted in (1) a positive (+), negative (−), variable (*σ*), or no change (0) in phytoplankton‐related variables when the response could be directional (e.g., increase in biomass), or (2) a change (∆) or no change (0) when the response could not be directional (e.g., change in functional composition). “na” indicates not applicable. The number of links may be greater than the number of storm events as a single storm may have multiple physical and chemical pathways (links) to a phytoplankton‐related variable. * indicates biomass or any other quantification of phytoplankton abundance different from chlorophyll. # indicates production or any other rate processes such as nutrient uptake rates

We classified the influence of storms on the physics and chemistry of the water bodies into six different lake condition variables (Figure 1; Table 1): (a) *hydrology*, related to many processes (e.g., flushing rates, floods, runoff, water level fluctuation, dilution, etc.); (b) water *temperature* at any depth in the water column; (c) *thermocline* depth; (d) *light* conditions, related also to water turbidity; (e) *nutrients*, including a variety of elements such as phosphorus, nitrogen, and silica; and (f) *mixing* (changes in thermal stratification). We also classified the effects of storms on phytoplankton into eight variables to assess research focus areas (Figure 1; Table 1): (a) *spatial displacement* (changes in the horizontal or vertical position); (b) *algal blooms* (especially changes in the frequency or prevalence of cyanobacterial blooms); (c) *biomass*; (d) *chlorophyll a*; (e) *production* (or any other rate processes such as nutrient uptake rates); (f) *community composition* (changes in the abundance of particular taxa); (g) *functional composition* (changes in any trait or function such as cell size or using any form of functional classification); and (h) *diversity* (including variables such as taxonomic richness or diversity indices).

One of the 31 studies (Yang et al., 2016) accounted for 81% of all storm events (Table 1); we did not include this study in the following summary of the literature because of its overwhelming influence (see Box 1). Two papers by the same authors (Pannard, Bormans, & Lagadeuc, 2007, 2008) assessed the same storms on the same two lakes, and thus were combined to represent two studies instead of four (Table 1). The 28 studies reported 77 storm events, of which 44% were related to wind, 33% to precipitation, and 23% to combined wind plus rain. In general, the relationship between storms and lake conditions were as expected—light and temperature decreased and mixing increased (Table 1). Nutrients increased and thermocline deepened, but both also showed variable responses. The hydrology of lakes, when reported, typically changed in response to rain and was associated with system flushing (Table 1).

**Table 1:**
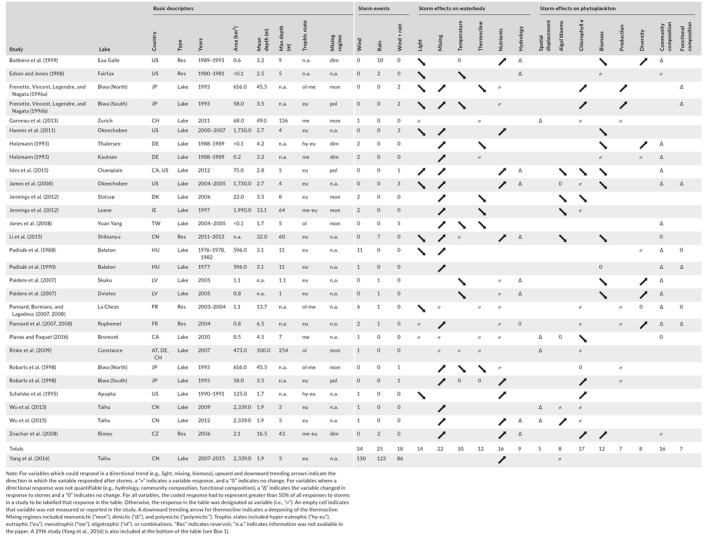
Summary of 28 studies that met criteria for links of (a) storm effects on (b) physics/chemistry with responses by (c) phytoplankton in lakes, reservoirs, or ponds

BOX 1 **What is “extreme” anyway?**

Studies from our systematic review varied greatly in how storm events were conceptualized, enumerated, and analyzed. For example, Yang et al. (2016) identified 339 extreme weather events (EWE) over a 9 year period on Lake Taihu, China, whereas Barbiero, James, and Barko (1999) identified 10 disturbance events over a 5 year period on Eau Galle Reservoir, United States (Table 1). For illustrative purposes, we use Yang et al. (2016) to highlight several open‐ended questions as key considerations for future studies of the impacts of storms on lake ecosystems.

**Are responses to storms extreme in both space and time?**

No single definition exists as to what constitutes an “extreme” biological response. However, in the case of cyanobacterial blooms, Yang et al. (2016) highlight that such definitions may include a spatial dimension, rather than solely a temporal dimension. They quantified the magnitude of cyanobacterial blooms based on temporal variation in the spatial extent of blooms using satellite‐derived data, rather than temporal variation in cyanobacteria at a single sampling point. Identifying the space and timescales of the extreme phenomena under investigation with storms is an important consideration going forward.

**Forward and reverse mapping of EWEs and biological responses**

Yang et al. (2016) illustrate an important feature of event‐based analysis to evaluate cause and effect—how we connect extremes in drivers and responses. They achieved this connection in a reverse direction: “extended” (i.e., extreme) cyanobacterial blooms were first identified (the effect) and then an antecedent period was searched for an EWE (the putative cause). The alternative is to first determine the timing of each EWE (the cause), and then search a subsequent time period for the incidence of an extreme response (the effect). The approaches answer different questions—how many extreme blooms might be driven by extreme weather, versus how many EWE precede extreme blooms? Yang et al. (2016) determined that approximately half (47/93) of their extreme blooms were potentially linked to extreme weather in the preceding time period. In a broader sense, what proportion of weather and bloom extremes need to temporally coincide to constitute strong evidence for cause and effect?

**Frequency of “extremes”**

By definition, extreme ecological conditions are state and process variations beyond “normal” system behavior and thus are rare. One approach to discern what constitutes an extreme event is to establish scientifically robust thresholds beyond which observations are considered extreme. Yang et al. (2016) adopted such an approach by defining EWEs as conditions in which daily average wind speed and rainfall exceed 4 m/s and 20 mm, respectively, and extended blooms as those >300 km^2^. These thresholds yielded 339 EWEs (see their figure 6a) and 93 satellite‐determined extended (i.e., extreme) blooms over their 9‐year study. Important questions to consider include the frequency of occurrence of bloom events relative to EWEs. If one changed the threshold weather conditions to define EWEs or extreme bloom conditions, one would also change the number of events detected and linked. When lake ecosystems are frequently disturbed by stormy weather, their communities likely comprise species that are well‐adapted to such conditions. What are the necessary considerations to set thresholds that ensure nontypical events are EWEs, and that they are beyond the range that resident biota normally experience? Recent work on the “tailedness” of biological and environmental variables, and their relationships in the context of extreme events, may prove a useful approach to future studies of the impacts of storms on lake ecosystems (Batt, Carpenter, & Ives, 2017).

To further explore the relationships among storms, lake condition variables, and phytoplankton described in our systematic review, we coded the number of times a storm was linked to a lake condition and then to a phytoplankton response. For example, if a study reported an effect of wind on mixing and then on phytoplankton biomass and community composition, this represented two three‐step links—the first connecting wind‐mixing‐biomass and the second connecting wind‐mixing‐community composition. A single storm could have multiple links with different lake and phytoplankton variables. Wind events were reported in 43% of the 28 studies, and were most commonly linked to changes in water column mixing (Figure 1a). All lake condition variables except hydrology were linked at least once to one of the eight phytoplankton variables, but only mixing was linked to all of them. Overall, the most frequent three‐step link for wind events was wind‐mixing‐chlorophyll *a* (30% of studies, *n* = 11), but the response of chlorophyll *a* to wind events was not consistent. Responses included all possible outcomes (increase, decrease, no change, or variable), with a variable response being the most common (Figure 1a). Of the categorical (change, no change, variable) phytoplankton‐related variables, change after wind events was much more common for community composition and spatial displacement, compared to a nearly equal split in responses for functional composition (Figure 1a).

Rain events were reported in 29% of studies although the number of links between rain events and lake condition variables (*n* = 74) was greater than that of wind events (*n* = 68; Figure 1b). Rain–hydrology, rain–temperature, and rain–nutrient were the most commonly described links of rain events to lake conditions, with relatively frequent connections to biomass and community composition. Phytoplankton biomass, when evaluated after rain events, was found to decrease in almost all cases, whereas diversity increased and community composition changed or remained the same with nearly equal frequencies (Figure 1b). Studies that included rain events did not evaluate or did not find many connections to spatial displacement, blooms, chlorophyll *a*, production or functional composition.

Finally, the combination of wind plus rain events was reported in 29% of the studies (*n* = 62 linked events). Links of wind plus rain events were found for all lake condition variables, with mixing (29% of studies) and nutrients (25% of studies) the most frequent (Figure 1c). Changes in lake conditions from wind plus rain events were most frequently related to chlorophyll *a* and production (21% and 18% of the studies), with few, if any, links to spatial displacement, blooms, or diversity (Figure 1c). In general, biomass decreased as a result of wind plus rain events, whereas production and chlorophyll *a* tended to increase or have a variable response. Community and functional compositions both changed after all wind plus rain events evaluated in the studies that met our criteria (Figure 1c).

Overall, our systematic review suggests variable effects of storms on phytoplankton. Biomass was the only phytoplankton variable that consistently responded (decreased) to all three types of storm events. Diversity consistently increased with rain events, community and functional compositions consistently changed with wind plus rain events, and spatial displacement was consistently evident with wind events, but all four of these phytoplankton variables responded inconsistently to other types of storm events (Figure 1; Table 1). The responses of the remaining phytoplankton variables, in general, were distributed across all possible responses with no clear patterns. The observed discrepancies of responses across and within phytoplankton variables and types of storm events suggest some level of context‐dependency in the effects of storms on phytoplankton, and support the need for a conceptual framework to navigate within such complexity.

The need for a conceptual framework is further supported by the relatively low sample sizes on which our review is based. Few studies have examined the full causal chain from meteorological forcing on lake conditions, mediated by lake and watershed characteristics, to the resultant response(s) of phytoplankton communities. In particular, half of the wind events in our review came from only three studies (Padisák et al., 1988; Pannard, Bormans, & Lagadeuc, 2007; 2008) and 40% of the rain events came from one study on a single reservoir (Barbiero et al., 1999). Furthermore, our conclusions may be influenced by sampling frequency and the duration over which effects were examined (Table S3). Twenty‐five percent of the studies sampled phytoplankton at daily or subdaily frequencies and 75% sampled every 2 days to 2 weeks (studies often sampled at multiple frequencies), while the duration over which impacts were evaluated ranged from within a day to multiple years (Table S3). The literature was also biased toward northern temperate and productive systems, reflecting the need to study (a) tropical regions that often deviate in mixing regime from temperate lakes, typically due to an increased importance of hydrological forcing (de Senerpont Domis et al., 2013; Sarmento, Amado, & Descy, 2013); and (b) meso‐, oligo‐, and dystrophic systems. Moreover, the definition of “storm” in limnology is inconsistent, when defined at all, and phytoplankton response variables are typically state‐ rather than process‐based. Clearly, our understanding of ecosystem‐scale responses of lakes to storm events is fragmented, and great terminological variability among studies hinders the resolution of generalizable impacts.

Within this context, and to help shape a limnological definition of “storm” separate from the meteorological definition given above (sensu World Meteorological Organization, 1967), we consider storms as discrete disturbance events generated by meteorological forcing that result in abrupt changes in the physical and/or chemical states of lakes relative to baseline background levels (Jennings et al., 2012). Our interest lies in the effects of storms on the primary resources for phytoplankton in lakes (i.e., light and nutrients) and water temperature, and how phytoplankton community structure and traits lead to resistance, resilience, temporal variability, and recovery under such abrupt changes, and thus is embedded in the general frameworks of disturbance and stability.

## 3 LAKE AND WATERSHED ATTRIBUTES AS MEDIATORS OF STORM IMPACTS

**FIGURE 2:**
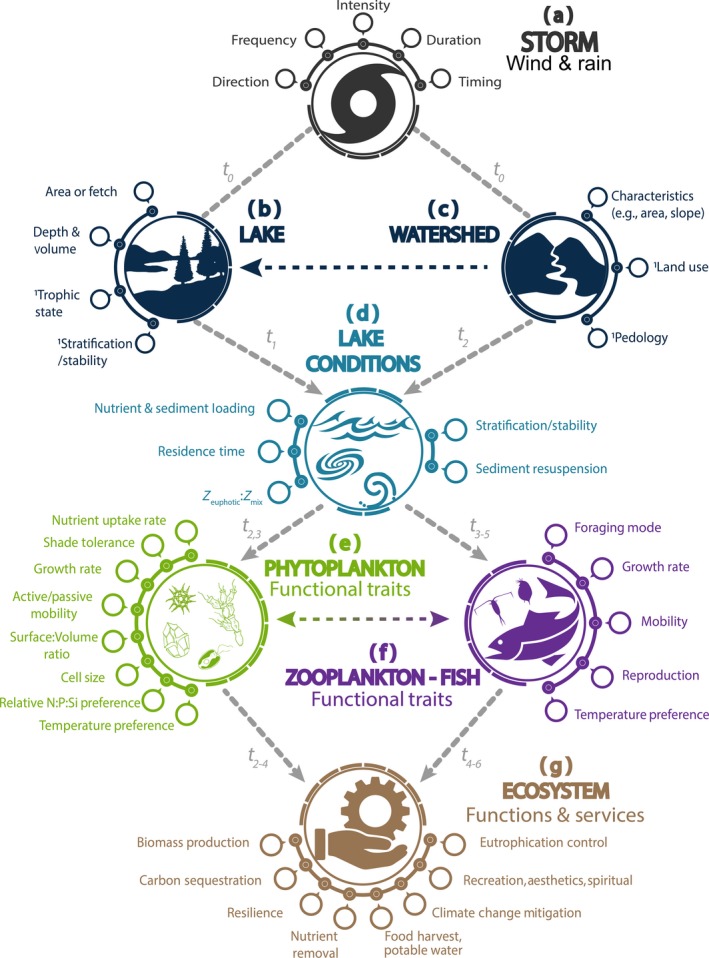
Conceptual model of how storm (a), lake (b), and watershed (c) attributes, and antecedent conditions, combine to alter light and nutrient conditions of lakes (d), with examples of phytoplankton (e) and higher trophic level (f) functional traits which likely play important roles in phytoplankton competition for survival and growth after storm‐induced disturbances, and ultimately ecosystem functions and services (g). However, details on the interactions of higher trophic levels and ecosystem functions and services in relation to storm impacts on phytoplankton is beyond the scope of this paper. Superscript^1^ indicates the role antecedent conditions may play in mediating the effects of storms on the lake ecosystem. Responses of lake ecosystem components to direct and indirect storm impacts manifest over variable timescales and lags, as indicated by *t*_0_ to *t*_6_, and response trajectories may not be linear; *t*_0_—immediate impact; *t*_1_ to *t*_6_—increasing timescales from hours to possibly decades

The same storm will impact different lakes in different ways (Klug et al., 2012; Kuha et al., 2016), and the same lake will respond to different storms in different ways depending on antecedent conditions (see below) and the incidence of compound climatic events (Leonard et al., 2014; Perga et al., 2018). Consequently, “strength” of a storm is necessary but not sufficient to understand the degree to which storms impact light, nutrients, and temperature and thus phytoplankton community dynamics. Lake and watershed attributes are key in mediating the extent to which a storm will impact lake conditions (Figure 2).

### 3.1 Wind‐induced lake mixing (Path a→b→d, Figure 2)

While wind forcing clearly impacts three‐dimensional circulation patterns in lakes, we focus on the vertical structure of the water column; physicochemical environmental gradients are especially pronounced in this dimension, and stand to be greatly modified by storm‐driven mixing events. Lake area and orientation (i.e., fetch) interact with wind speed and direction to influence mixing (Fee, Hecky, Kasian, & Cruikshank, 1996; Hondzo & Stefan, 1993; Read et al., 2011), and determine water column effects such as internal waves, upwelling, thermocline and mixing depths, photic zone temperature, and sediment/nutrient resuspension (Hamilton & Mitchell, 1996; Horn, Mortimer, & Schwab, 1986; Søndergaard, Kristensen, & Jeppesen, 1992; Figure 2a–c). Such processes are important, but have different effects in deep and shallow water bodies.

**FIGURE 3:**
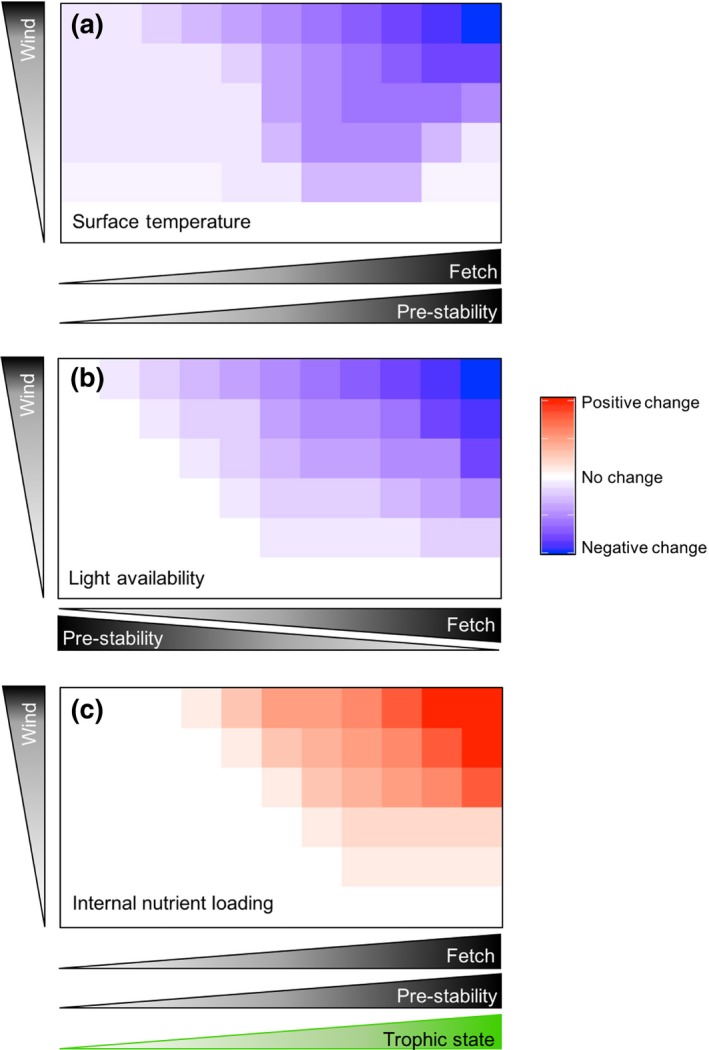
Impacts of wind events on surface water temperature, light availability, and internal nutrient loading are expected to be mediated by lake fetch, antecedent (“pre”) water column stability, and trophic state. As momentum and mechanical energy flux across the lake–air interface, they scale as the wind speed squared and cubed, respectively (Wüest & Lorke, 2003). Thus, even relatively modest increases in wind speed could lead to disproportionately large changes in lake stratification and mixing dynamics. Lake depth also plays a role in mediating the impacts of wind events (see Figure S1). (a) In general, if a lake is stratified, wind will deepen the upper mixed layer, increase the volume of water within the upper layer, and thus reduce surface temperature. Polymictic lakes (lower prestability) still tend to have cooler temperatures at depth and the same processes could be important in altering surface temperatures, albeit to a lesser extent. Strong antecedent stability is characterized by sharp temperature gradients and resistance to mixing, but such conditions also set the stage for the greatest change in surface temperature. For example, if stability and wind speed are high, we expect a seiche to develop with the potential for upwelling of cold, hypolimnetic waters to the lake surface. (b) Wind events on lakes with weaker antecedent water column stability and greater fetch will have larger negative effects on light availability than on lakes with stronger antecedent stability and shorter fetch. (c) Wind events are expected to have the greatest impact on internal nutrient loading in lakes with greater fetch, stronger antecedent stability, and higher productivity. In particular, strong antecedent stability is expected to facilitate the buildup of nutrients in hypolimnetic waters (deeper lakes) and nutrient release through sediment anoxia (shallower lakes; see Figure S1 for more details), although well‐oxygenated hypolimnia likely result in little effect

Larger lakes typically experience higher wind speeds than smaller lakes because of longer fetch (Docquier, Thiery, Lhermitte, & van Lipzig, 2016; Hondzo & Stefan, 1993), and are likely to experience stronger wind‐induced mixing (Fee et al., 1996; Kling, 1988; Patalas, 1984; Read et al., 2011). Thus, in general, we expect wind impacts on lake surface temperatures, light availability for phytoplankton, and internal nutrient loading to increase with increasing surface area (as an important component of fetch; Figure 3), but the impacts are mediated by lake depth (Figure S1).

The average temperature of the mixed layer can drop during a storm depending on lake‐specific characteristics and the strength of the storm (Jennings et al., 2012; Kuha et al., 2016; Woolway et al., 2018)—a result of the deepening of the mixed layer and the entrainment of colder metalimnetic and hypolimnetic waters, or by internal waves breaking on the bottom (Kasprzak et al., 2017; Pöschke et al., 2015; Schladow, Pálmarsson, Steissberg, Hook, & Prata, 2004). Surface cooling may also increase wind‐induced mixing during storms, as overcast conditions and colder air temperatures often coincide with storm events (Jennings et al., 2012). Decreased water column stability as a result of wind‐induced mixing will increase mixing depth (*Z*_mix_) relative to the euphotic depth (*Z*_eu_) and thus reduce *Z*_eu_:*Z*_mix_ and the effective daylength (i.e., mean light intensity and distribution of light intensities) experienced by phytoplankton (Fee et al., 1996; Litchman, 2000; MacIntyre, 1993; Reynolds, 1994; Shatwell, Nicklisch, & Köhler, 2012).

Lakes with high surface area (and fetch) may also be associated with higher sediment resuspension, particularly in shallow lakes and in the littoral zone of deeper lakes, which will further impact light availability through a reduction in *Z*_eu_ (Padisák et al., 1988; Figure S1). Additionally, storms can uproot macrophytes in shallow lakes and littoral zones of deep lakes, leading to turbidity which prevents macrophyte regrowth and alters the competitive playing field of primary producers (Hilt, 2015; Schutten & Davy, 2000; Williams, 1979). One exception where wind‐induced mixing from storms may partially alleviate light limitation is the entrainment of deep chlorophyll maxima into surface waters as a result of upwelling, particularly in clear lakes with extended pelagic zones (Kasprzak et al., 2017), although this may be offset by downwelling in other parts of the lake.

Nutrient renewal from deeper waters and/or sediments to the euphotic zone (i.e., internal loading) can also result from wind‐induced mixing and upwelling (Figure 3; Carper & Bachmann, 1984; O'Reilly, Alin, Plisnier, Cohen, & McKee, 2003; Verburg, Hecky, & Kling, 2003; Wilhelm & Adrian, 2008). Thus, we expect nutrient resuspension as a result of wind‐induced mixing to be positively correlated with fetch (Figure 3), although lake depth also plays a role (Figure S1; MacIntyre et al., 2006). In stratified lakes, internal waves and wind‐induced tilting of the thermocline can lead to the upwelling of hypolimnetic waters with relatively high nutrient concentrations, especially in eutrophic water bodies (Gächter & Wehrli, 1998; Soranno, Carpenter, & Lathrop, 1997), and internal loading from the sediment versus the hypolimnion can be an important distinction (Wilhelm & Adrian, 2008). The N:P ratio of nutrients brought to the surface by wind‐induced mixing is often low as a result of denitrification at the water–sediment interface or in anoxic hypolimnia (Huber, Wagner, Gerten, & Adrian, 2012), and can lead to favorable conditions for N‐fixing cyanobacteria (Wagner & Adrian, 2011). In shallow lakes where the surface sediment is often well oxidized, aerobic release of phosphorus can be substantial (Jensen & Andersen, 1992). However, low oxygen in bottom waters of shallow lakes can enhance internal loading from the sediment (de Senerpont Domis et al., 2013; Gerling et al., 2016; Wilhelm & Adrian, 2008). Deep lakes typically experience less internal loading from the sediment because of lower nutrient concentrations and lower hypolimnetic oxygen consumption rates (Wetzel, 2001), although sediment oxygen demand can be high in deep meso‐eutrophic lakes leading to high rates of phosphorus release (Prepas & Burke, 1997).

### 3.2 Precipitation‐induced nutrient and sediment loading, light limitation, and temperature cooling (Paths a→c→d and a→c→b→d, Figure 2)

**FIGURE 4:**
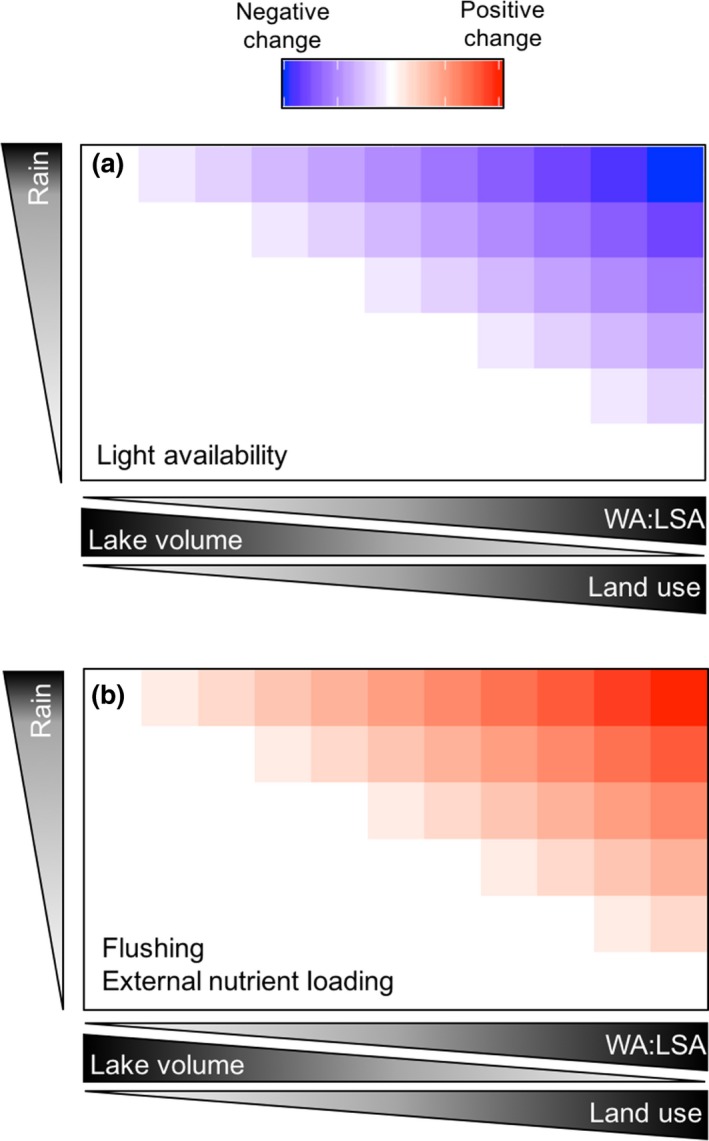
Impacts of precipitation events on light availability, system flushing, and external nutrient loading are expected to be mediated by lake and watershed attributes that include ratio of watershed area to lake surface area (WA:LSA), lake volume, and anthropogenic land use (e.g., urban or agricultural development). (a) Sediment and dissolved organic carbon delivered to lakes by runoff from precipitation will reduce light availability (penetration) in lakes. We expect that light availability to phytoplankton will be more negatively impacted as WA:LSA and anthropogenic land use increase and lake volume decreases (Figure S2). (b) Flushing rates of lake systems as a result of precipitation runoff will be greatest in lakes with large WA:LSA, more anthropogenic land use, and small lake volumes. Lakes with large volumes, relatively small watershed areas, and less developed landscapes will be more buffered from precipitation‐induced flushing. We expect similar patterns for external nutrient loading. In particular, external nutrient loads will be diluted in lakes with larger volumes, and therefore are less impacted by precipitation events, at least in the short term. Long‐term buildup of external nutrient loads can eventually lead to excessive internal nutrient loading (Figures S1 and S2)

The interactions of lake morphology with watershed attributes are also important for how lakes respond to rain events (Figure 2a–d). Precipitation is the primary driver of watershed‐mediated storm impacts on lakes. The ratio of watershed area to lake surface area (WA:LSA) is an indicator of external water, nutrient, and sediment loads and how much they may affect a lake (Gergel, Turner, & Kratz, 1999; Knoll et al., 2015). In general, the higher the WA:LSA, the larger the impact storm runoff has on lake conditions (Shen, Koch, & Obeysekera, 1990). In particular, we expect that the impacts of precipitation on external loading, changes in light availability, and system flushing (i.e., hydraulic residence time) will be positively related to WA:LSA (Figure 4; Figure S2; Sobek, Tranvik, Prairie, Kortelainen, & Cole, 2007). However, the influence of precipitation events will also be mediated by other aspects of both watershed and lake morphology such as land use and cover, the degree of connectivity with headwaters, watershed slope, soil properties, drainage density, waterbody origin, and lake volume (Figure 2b,c). For example, canopy cover and soil percolation differ among forested, urbanized, and agricultural watersheds and can modify external loads into a lake (Carpenter et al., 1998; Fraterrigo & Downing, 2008; Hall, Leavitt, Quinlan, Dixit, & Smol, 1999). Both sediment load and composition will have important impacts on light attenuation and nutrient concentrations within lakes. Sediment loading from runoff can decrease the euphotic zone (Havens, James, East, & Smith, 2003; Søndergaard, Jensen, & Jeppesen, 2003) but increase short‐ (i.e., external loading) and long‐term (i.e., future internal loading) nutrient availability, depending on the form(s) in which nutrients are delivered to the lake (i.e., particulate/dissolved, labile/recalcitrant and inorganic/organic; Hayes, Vanni, Horgan, & Renwick, 2015; Robson & Hamilton, 2003). Rapid increases in lake water level due to an extreme precipitation event may flood previously exposed lake beds, impacting first the littoral zone, and then with cascading impact on phytoplankton (Jeppesen et al., 2015; Zohary & Ostrovsky, 2011). Consequently, we expect the impacts of precipitation on external loading, changes in light availability as a result of sediment loads, and system flushing to be positively related to the degree of anthropogenic land use in a watershed (Figure 4; Figure S2). In extreme cases, system flushing could potentially counteract increased external loading by flushing nutrients out of the system.

Compared to other meteorological variables known to influence the surface temperature of lakes (Edinger, Duttweiler, & Geyer, 1968), the influence of precipitation on the lake surface temperature is relatively unexplored. One exception is the study of Rooney, van Lipzig, and Thiery (2018), who demonstrated that in tropical Lake Kivu, the surface temperatures cooled by ~0.3°C as a result of precipitation. The change was explained, in part, by the influence of precipitation on (a) the surface heat budget via the rain heat flux (where the rain is cooler than the lake surface temperature); and (b) its influence on surface mixing both directly through enhanced kinetic energy and indirectly by modifying convective mixing in the surface layer (Rooney et al., 2018). Turbidity also plays a role in water temperature, as suspended solids in water absorb and scatter sunlight, with turbid near‐surface water layers warmer than clear near‐surface water layers (Paaijmans, Takken, Githeko, & Jacobs, 2008). Precipitation runoff leading to increases in turbidity could therefore lead to higher water temperatures.

Waterbody origin influences the sensitivity of a waterbody to storm impacts. Because of their much higher WA:LSA than glacially formed lakes (Doubek & Carey, 2017), reservoirs may receive disproportionately more sediment loading than lakes for a given watershed size due to their riverine and dendritic nature (Knoll et al., 2015; Thornton, 1990; Whittier, Larsen, Peterson, & Kincaid, 2002; Figure S2d). However, reservoirs generally have shorter hydraulic residence times and faster flushing rates than natural lakes, especially those built by impounding lotic systems (Doubek & Carey, 2017; Wetzel, 1990). Therefore, impacts of external loading into reservoirs could be shorter‐lived relative to natural lakes, as nutrients and sediments brought into reservoirs are often flushed out more quickly than in natural lakes (Figure S2). Similarly, smaller and shallower lakes with surface inflows and outlets will be more prone to rapid storm flushing as many have hydraulic residence times of weeks to months, compared to larger and deeper lakes which can have residence times of decades to centuries (Figure S2). Impacts of external loading and changes to *Z*_eu_:*Z*_mix_ as a result of runoff are expected to be lower in lakes with larger volumes through a dilution effect (Figure S2; Scheffer & van Nes, 2007). In short, the extent to which a lake's environmental conditions are affected by storm events will be highly mediated by many attributes intrinsic to the lake and its watershed, and not just by the storm itself (Figure 2).

### 3.3 Antecedent conditions (Paths a→b→d, a→c→d, and a→c→b→d, Figure 2)

Antecedent conditions in lakes and their watersheds, such as soil frost and lake ice, thermal stratification, soil conditions, and land use (Figure 2b,c), can further influence effects of wind and precipitation (Figure 2a) on in‐lake light, nutrient, and temperature conditions (Figure 2d). For example, extended periods of low wind or warm weather strengthen thermal stratification (Huber et al., 2012). Strongly stratified water columns are less likely to become fully mixed after a wind event (Abbott et al., 1984; Gorham & Boyce, 1989) and are likely to return more quickly to fully stratified conditions than weakly stratified water columns (Magee & Wu, 2017; Woolway, Meinson, Nõges, Jones, & Laas, 2017; Woolway & Simpson, 2017). Thus, we expect a negative relationship between storm‐induced water column mixing and the strength of stratification prior to the wind event (Figure S1), although the susceptibility of the water column to mixing can increase after each wind event depending on the frequency of occurrence and magnitude of such events (Churchill & Kerfoot, 2007). Changes in light availability (Huisman, van Oostveen, & Weissing, 1999) resulting from storm‐induced mixing will also likely be negatively related to the strength of antecedent stratification, as will the potential for sediment resuspension (Figure S1). Strong stratification can also promote nutrient depletion in the mixed surface layer (Reynolds, 1976; Round, 1971; Verburg et al., 2003; Wilhelm & Adrian, 2008) and nutrient accumulation in hypolimnetic waters (Reynolds, 1980; Sommer, Gliwicz, Lampert, & Duncan, 1986; Søndergaard et al., 2003), which could influence the change in nutrients in the photic zone when nutrients are translocated from the hypolimnion as a result of storm events (Figure S1). However, we expect internal loading to the photic zone to be negatively related to stratification strength prior to a storm event because of the increased resistance to mixing.

Similarly, antecedent conditions related to weather, soil, and land use can greatly influence external nutrient and sediment loading to lakes. For example, extended warm, dry weather can promote buildup of glacial flour through glacial abrasion and lead to increased glacial flour runoff with subsequent rain events, which can alter the light environment and thermal stratification in lake systems (Collins, 1989; Perga et al., 2018). In cold climates, precipitation in the form of rain or snow will likely have varying impacts on lakes in terms of immediate versus lagged effects depending on lake and watershed conditions (e.g., ice, snowpack, frozen soil; Johnson & Stefan, 2006; Joung et al., 2017). Weather conditions also influence land use practices, including the type and amount of fertilizer applied in agricultural watersheds, which can cause excessive nutrient loading depending on timing, intensity, and duration of precipitation events (Michalak et al., 2013). Consequently, the timing of storms in relation to antecedent conditions in lakes and their watersheds play an important role in the effects of storm‐induced mixing and external loading on resource dynamics for phytoplankton communities (Andersen et al., 2006; Mi, Frassl, Boehrer, & Rinke, 2018; Roozen et al., 2008).

### 3.4 Phytoplankton traits as mediators of storm impacts (Path d→e, Figure 2)

The lake conditions which emerge after a storm event create the stage on which phytoplankton community dynamics play out (Figures 2, 3, 4). Phytoplankton taxa are differentially adapted to a range of environmental conditions (Litchman & Klausmeier, 2008; Reynolds, 2006). Changes in light and nutrient availability and thermal conditions may affect resource competition (Chesson, 2000; Ptacnik, Moorthi, & Hillebrand, 2010) based on physiological characteristics such as nutrient uptake and storage capacity, buoyancy regulation, and partitioning of absorbed light energy (Table 2; Figure 2e; Fanesi, Wagner, Becker, & Wilhelm, 2016; Reynolds et al., 2002; Salmaso et al., 2015). However, such changes are usually embedded within seasonal variation in light and nutrient limitation that mediate their biological effects, especially in temperate and high‐latitude lakes. For example, autumn wind and rain events in temperate reservoirs pushed the phytoplankton community toward diatom dominance by disrupting stratification and increasing external and internal nutrient loads (Pannard et al., 2008). However, diatoms declined after similar storm events in late spring, when nutrients were abundant and increased flushing rates favored species with higher maximum growth rates (Pannard et al., 2008). During the stratified period, the phytoplankton community may show resistance to perturbation and high poststorm resilience in deep lakes with strong prestorm stratification (Holzmann, 1993). In other words, lake attributes and antecedent conditions that increase physical resistance to storms may increase biological resistance as well.

**Table 2:** Expected associations between functional traits of freshwater phytoplankton and abiotic variables associated with potential storm effects in lakes

	Phytoplankton functional traits						
	Flagella/motility (controlled vertical migration)	Small cell size (rapid growth, slow settling)	Spherical colonies (nutrient acquisition, grazing resistance)	Filamentous (light capturing efficiency)	Gas‐vesicles/mucilage (buoyancy regulation/controlled vertical migration)	Silicaceous (silica‐limited; rapid sinking)	N_2_‐fixation
Example taxa	*Cryptomonas marsonnii* (Cryptophyceae)	*Cyclotella* spp. (diatoms)	*Volvox* spp. (green algae)	*Planktothrix agardhii* (Cyanobacteria), *Mougeotia* spp. (green algae)	*Microcystis*, *Planktothrix rubescens* (Cyanobacteria)	*Aulacoseira* spp. (diatoms)	*Aphanizomenon* spp. (Cyanobacteria)
Example references	Jones (1988), Clegg, Maberly, and Jones (2003), Salonen, Jones, and Arvola (1984)	Rühland et al. (2015)*	Reynolds, Wiseman, and Clarke (1984)	Scheffer, Rinaldi, Gragnani, Mur, and van Nes (1997), Reynolds et al. (2002)	Reynolds, Oliver, and Walsby (1987)*	Rühland et al. (2015)*	Paerl and Otten (2013)
Abiotic variables							
Nutrient loading (internal or external)	−	+	−	±	±	+	±
Decreased *Z*_eu_/*Z*_mix_	+	−	+	+	+	+	−
Flushing	−	+	−	+	−	−	−
Low temperature	−	+	−	−	−	+	−
Turbulence/mixing strength	−	+	−	±	−	+	−
Stable, stratified environment	+	−	+	−	+	−	+

Note

A “+” indicates a generally positive association (the trait becomes more dominant after a physical storm effect), while “−” indicates a generally negative association. A “±” indicates the possibility of positive or negative association, depending on antecedent conditions. Changes in trait dominance within the phytoplankton community reflects trait variation within a taxonomic group as well as turnover among groups (Litchman & Klausmeier, 2008). The physiological/ecological functions of each trait are given in parentheses (derived from Salmaso et al., 2015). Expected associations, and example genera or species that exhibit each trait, were derived from the cited references, and may not be universally applicable. The realized environmental tolerances of a species are subject to the simultaneous influence of multiple traits (Litchman et al., 2010). An “*” indicates a literature review.

To further complicate the picture, storm effects often manifest through multiple mechanistic pathways with contrasting implications for phytoplankton. For example, our systematic review indicates that wind storms can simultaneously reduce light availability while increasing nutrient concentrations in the water column through sediment resuspension and mixing (Table 1). This type of resource trade‐off appears common, and has been documented in reservoirs and natural lakes, oligotrophic to eutrophic systems, and temperate to tropical latitudes (Frenette et al., 1996a; James et al., 2008; Pannard et al., 2007, 2008).

Phytoplankton species are not physiologically adapted to all conditions, and trade‐offs among physiological traits are essential to understand phytoplankton community responses to such complex storm effects (Litchman, Klausmeier, Schofield, & Falkowski, 2007). For example, summer typhoons in Lake Biwa disrupted stratification and stirred up sediment (Frenette et al., 1996a, 1996b). The resulting decrease in light and increase in suspended phosphorus favored large‐celled species that grew relatively quickly in low light and had the capacity to rapidly take up and store nutrients (Frenette et al., 1996b). The dominance of large‐celled, yet fast‐growing species led to a net increase in total phytoplankton biomass compared to prestorm conditions (Frenette et al., 1996b). Similarly, hurricanes in tropical Lake Okeechobee induced a shift from a community dominated by cyanobacteria adapted for nutrient‐limited and high‐light stratified environments toward a poststorm community comprised mainly of low‐light tolerant diatoms (James et al., 2008). This pattern of increased abundance of large‐celled diatoms coupled with a decline in colonial cyanobacteria has been reported in a variety of systems and can be explained by a trade‐off among physiological traits that represent adaptations for light versus nutrients (Pannard et al., 2008).

Despite the conceptual appeal of trait‐based approaches, they can be difficult to apply. In particular, the traits or physiological characteristics that are relevant to a particular research question are not always clear, and traits such as maximum growth rates and light use efficiency can be difficult to quantify (Funk et al., 2017). However, physiological tolerances are constrained by morphological traits such as cell size, surface to volume ratio, and growth form, such that morphological measurements can be used to derive expectations for relationships among environmental conditions and dominant phytoplankton morphologies (Table 2; Litchman & Klausmeier, 2008; Litchman et al., 2007). Although trait expression is often plastic (e.g., some species occur as colonies and as single cells), most taxa can be reasonably characterized by their average functional traits (Litchman & Klausmeier, 2008). Trait variation within communities can therefore be summarized by sorting phytoplankton species into functional groups based on patterns of co‐occurrence in so‐called morpho‐functional traits (morphological characteristics that influence ecological function; Kruk et al., 2010; Reynolds, 1988b; 2002; Salmaso & Padisák, 2007; Salmaso et al., 2015).

Among existing functional group classifications, the C‐S‐R model (Reynolds, 1988b) stands out as a compelling framework for linking storm events with phytoplankton community dynamics. Reynolds reasoned that phytoplankton can be meaningfully divided into three groups, based on their distribution among orthogonal gradients of light and nutrients: Competitors that thrive under abundant light and nutrients, Stress‐tolerant species adapted to nutrient scarcity, which can occur during prolonged stratification, and “disturbance‐tolerant” Ruderals (sensu Grime, 1979) that are adapted to frequently/strongly mixed conditions (Reynolds, 1988b). Because mixing events reduce light availability (Köhler, Wang, Guislain, & Shatwell, 2018), they can be considered a form of disturbance, and the C‐S‐R model provides testable hypotheses about the effects of high disturbance frequency and intensity on phytoplankton communities (R > C > S; Lindenschmidt & Chorus, 1998). C‐S‐R strategies also link light and nutrients with phytoplankton size and shape. Cell and colony size constrain metabolism, nutrient acquisition rates, and nutrient storage capacity, while elongate (filamentous) shapes naturally orient perpendicular to the underwater light gradient and maximize light‐capturing surface area, which makes these elongate shapes more adaptive under light limitation (Naselli‐Flores, 2014; Padisák, 2004; Reynolds, 1984, 1988b, 2006). These relationships imply that species‐specific responses to storm effects (e.g., nutrient subsidies from rain/flooding, or mixing below the euphotic zone) are predictable based on morphology.

**FIGURE 5:**
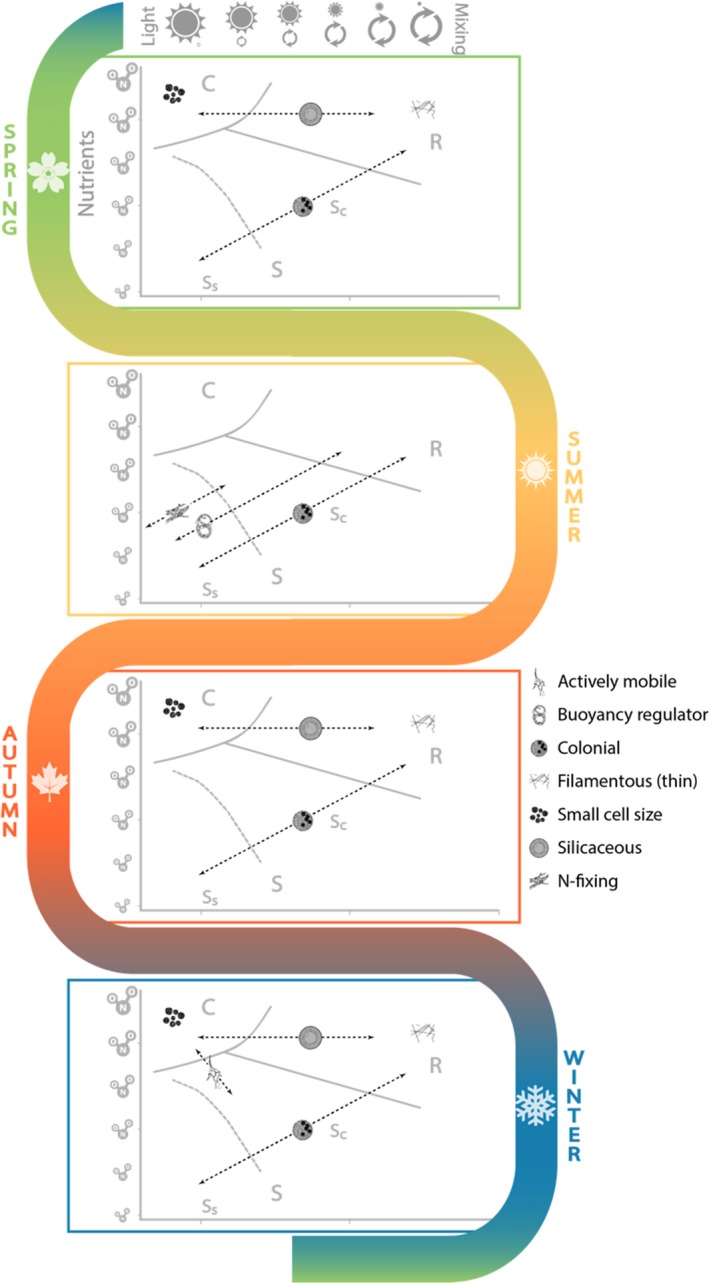
Seasonal mapping of morpho‐functional traits (see legend and Table 2) and C‐S‐R strategies as a function of environmental conditions susceptible to storm‐induced modification (modified from Madgwick, Jones, Thackeray, Elliott, & Miller, 2006). Dashed arrows represent the range of light/mixing (*x*‐axis) and nutrient (represented as NO_2_ in this case, *y*‐axis) conditions a functional trait could span. The seasonal plots are derived from temperature‐dependent growth of phytoplankton groups associated with each trait (Paerl & Otten, 2013)

Although its logic and simplicity are appealing, C‐S‐R alone may not fully capture storm‐functional trait relationships; arguably the limited number of groups in this model does not reveal the full extent of phytoplankton functional diversity. This limitation could be overcome by combining C‐S‐R with a more nuanced classification scheme, such as the morpho‐functional group (MFG) system (Salmaso et al., 2015), which uses a combination of taxonomy and morphological traits to identify ecologically relevant divisions within broad taxonomic groups such as the cyanobacteria, chlorophyceae, and diatoms. MFGs differentiate between, for example, large, colonial centric diatoms (MFG 6a1; e.g., *Aulacoseira*) that are dependent on turbulent resuspension, and small unicellular centric diatoms (MFG 7a; e.g., *Cyclotella*) that are better adapted for calm, nutrient‐rich conditions (Rühland, Paterson, & Smol, 2015), and can be conceptually mapped onto C‐S‐R strategies and light/nutrient gradients (Figure 5). Although they have not been widely applied to study storms, trait‐based functional classifications like MFG and C‐S‐R have been used to predict compositional shifts in phytoplankton communities in response to changes in nutrients, wind, rain, thermal stability, and thermocline structure over a variety of timescales (Abonyi et al., 2014; Deng, Salmaso, Jeppesen, Qin, & Zhang, 2019; Lofton, McClure, Chen, Little, & Carey, 2019; Tolotti, Thies, Nickus, & Psenner, 2012).

Functional group classifications can characterize biodiversity as well as community composition and provide insight into the mechanisms of diversity change. The intermediate disturbance hypothesis (IDH), which states that diversity is maximized at the intermediate intensity or frequency of disturbance (Connell, 1978), implicitly invokes the C‐S‐R concept (Weithoff, 2003). The IDH has been used extensively as a model for understanding phytoplankton community assembly patterns related to disturbance, including storm events (as described in depth in Padisák, Reynolds, & Sommer, 1993). For example, if the frequency or intensity of storm events is low, species that are most efficient in exploiting resources under stable environmental conditions will outcompete others, resulting in low phytoplankton diversity. If the frequency of storms is high, only phytoplankton species that can exploit well‐mixed environmental conditions or are within the dispersal range will persist. Thus, the phytoplankton diversity at the high disturbance extreme will depend on the interplay between disturbance and dispersal capabilities of the organisms (Altermatt, Schreiber, & Holyoak, 2011).

Unfortunately, while the frameworks described above account for the distribution of species along gradients in environmental conditions, they do not explicitly consider the role of antecedent conditions and seasonality. In the context of storm effects, antecedent conditions include the composition of prestorm phytoplankton communities as well as environmental variables such as trophic state (Padisák et al., 1993). For example, the response of cyanobacteria to increased nutrient loading can be positive or negative depending on functional traits, prestorm temperature, and nutrient availability, and whether inputs are dominated by nitrogen or phosphorus (Table 2; Ding, Qin, Deng, & Ma, 2017; Ding, Xu, Deng, Qin, & He, 2019).

To overcome this limitation, we conceptualize phytoplankton functional responses to the physical effects of storms as significant departures from typical “background” seasonal trait variation. For example, the temperature‐dependence and optima for phytoplankton growth vary among taxonomic groups associated with MFGs (e.g., chlorophytes, diatoms, cyanobacteria; Bergkemper, Stadler, & Weisse, 2018; Paerl & Otten, 2013). We can combine this knowledge with seasonal temperature limits for a hypothetical temperate lake, the traits associated with MFGs, and their position along the two axes of the C‐S‐R model to predict which MFGs are likely to dominate in each season under storm‐free light and nutrient conditions (Figure 5). Predicted prestorm trait distributions could then also be used to develop hypotheses about shifts in community composition in response to storm‐driven changes to light, nutrients, and stratification (Table 2). For example, small, rapidly‐growing unicellular chlorophytes and diatoms might be expected to dominate after rain storms that increase nutrient loads without reducing light, but these groups would be at a competitive disadvantage if such a storm occurred during high temperatures of summer (Figure 5). Hypotheses could be tested within a hierarchical modeling framework (e.g., structural equation modeling or Bayesian hierarchical linear models) that allow the strength of direct and indirect relationships between storm features and phytoplankton traits (Figure 2) to be quantified (Edwards Litchman, & Klausmeier, 2013a, 2013b; Lavorel & Grigulis, 2012; Pollock, Morris, & Vesk, 2012). Although the specific framework presented in Figure 5 is not valid for all taxa or lakes, this type of hierarchical modeling has been widely used to develop a trait‐based understanding of how environmental gradients affect species distributions and ecosystem functions in terrestrial, marine, and freshwater systems (Edwards et al., 2013a; 2013b; Lavorel & Grigulis, 2012; Pollock et al., 2012), and has great potential for testing hypotheses about the biological effects of storms in lakes.

We propose that phytoplankton diversity at high disturbance (storm) frequency will depend on antecedent conditions and storm intensity. Increasing storm intensity increases turbidity in meso‐eutrophic shallow lakes to levels that only a few taxa (e.g., mainly R‐strategists) can tolerate, leading to an overall decline in phytoplankton diversity. However, in deep stratified clear lakes, diversity might increase under the same storm pattern if the intensity and frequency increases heterogeneity in resource conditions (light and nutrients) without saturating the system with, for example, continual sediment resuspension. Future studies are needed to better understand how multiple factors such as storm intensity and lake antecedent conditions interact to affect phytoplankton diversity relationships.

## 4 RESEARCH DIRECTIONS

Our review demonstrates the need to integrate the multiple direct and indirect pathways by which storms impact watershed‐scale processes and in‐lake physics to drive abrupt changes in lake conditions, and the cascading impacts on lake biota. Such efforts are not only invaluable from a scientific point of view, but also crucial to control eutrophication and to optimize adaptive water resource management (Carpenter, Brock, Folke, van Nese, & Scheffer, 2015; Urrutia‐Cordero, Ekvall, & Hansson, 2016). To achieve robust aquatic ecosystem conservation and restoration in the context of climate change, knowledge of the extent to which storms impair ecological resilience is critical (Holling, 1973). Multidimensional approaches will be needed to better understand phytoplankton community responses to storms, where foci are placed on measuring multiple interrelated aspects of ecological stability (Hillebrand et al., 2018). Below we highlight several areas and approaches that will advance our understanding of storm impacts on phytoplankton communities, integrating data from a diverse range of spatial and temporal scales toward the goal of a better understanding of ecosystem resistance and resilience, critical in a time of rapidly changing climate.

### 4.1 The roles of lake and watershed attributes and antecedent conditions

The attributes of lakes and watersheds and their antecedent conditions play a large role in determining if meteorological storms translate into what may be called “limnological storms”—abrupt changes in the physical and/or chemical states of a lake relative to baseline levels (Jennings et al., 2012). The number of factors and processes involved in translating a meteorological storm to a response by the phytoplankton community are large and complex (Figure 2), but our review provides a framework to methodically examine how these processes and their interactions may translate into limnological storms. Future studies could use our conceptual model (Figure 2) based on a series of simple expectations of increasing/decreasing importance (Figures S1 and S2) to assess the response of lake conditions to storms as functions of lake‐specific attributes and antecedent conditions, and subsequent responses of phytoplankton communities using trait‐based expectations (Figure 5). Phytoplankton responses could be nonlinear, with synergistic or antagonistic effects on traits mediated by antecedent conditions. Such nonlinear responses may elucidate which attributes and antecedent conditions may be the most influential to phytoplankton community responses from storms (sensu van de Pol et al., 2017). Tracking single storms across a gradient of lake‐watershed types (Klug et al., 2012), comparing the response of multiple lake‐watershed types to localized storms (Jennings et al., 2012), or experimentally manipulating antecedent conditions (Flöder & Sommer, 1999) are promising approaches.

### 4.2 Integration of trait‐based and lake models

Trait‐based models are used to test hypotheses about population‐, community‐, and ecosystem‐level dynamics under the assumption that individual traits correlate with ecological function, and those individuals with functions best suited for current environmental conditions will be selected (Edwards, Thomas, Klausmeier, & Litchman, 2012; Violle et al., 2007). Such models perform well under stable or slowly changing conditions because selection pressures may be relatively consistent and trait variability or trait adaptation may not be important (Coutinho, Klauschies, & Gaedke, 2016; Merico, Bruggeman, & Wirtz, 2009; Weithoff & Beisner, 2019). The application of trait‐based models when abiotic conditions (i.e., selective pressures) change rapidly, such as during storm events, has to be further tested. Models that can reflect phenotypic plasticity and intraspecific variability at the timescale of disturbance events should be developed alongside our increasing understanding of how traits evolve over environmental gradients (Gaedke & Klauschies, 2017; Weithoff & Beisner, 2019). Species traits and growth parameters in models generally come from laboratory experiments, which might not represent the responses of organisms in natural settings, especially considering species dispersal. Because of this challenge, only a few lake models have been able to simulate emerging phytoplankton community dynamics in lakes (Janssen et al., 2015) and most models have been developed to provide estimates of algal biomass but not species composition and functional diversity. For example, the phytoplankton model PROTECH is able to reliably simulate C‐S‐R functional groups, and can be used in hypothesis‐testing frameworks for questions related to changes in phytoplankton communities from storms (Elliott, Irish, & Reynolds, 2001; Reynolds, Irish, & Elliott, 2001). If the next generation of lake models are to integrate multiple trait‐based approaches, more detailed and mechanistic phytoplankton modules will need to be developed to simulate functional diversity and dynamics (Mieleitner, Borsuk, Bürgi, & Reichert, 2008).

### 4.3 Integration of watershed and lake hydrodynamic models

Our synthesis demonstrates the importance to consider direct and indirect pathways by which storms impact lake ecosystems, which can be enabled by coupling watershed and lake models (Nielsen, Bolding, Hu, & Trolle, 2017). Coupled watershed and lake models have been used to simulate how changes in nutrient load and climate affect lake trophic state (Me, Hamilton, McBride, Abell, & Hicks, 2018), cyanobacterial dominance (Bucak et al., 2018), and chlorophyll concentration and dissolved oxygen levels (Crossman & Elliott, 2018). However, further technical development is required to overcome mismatches between model types, such as different timescales or different water quality constituents under simulation (Frassl et al., 2019), and to cascade uncertainties through the model chain. Extension of 1‐D lake models to 3‐D would provide a more complete simulation environment (Bocaniov, Ullmann, Rinke, Lamb, & Boehrer, 2014; Liu, Bocaniov, Lamb, & Smith, 2014; Soulignac et al., 2018) with which to couple trait‐based models (see above) to generate and test hypotheses on how meteorological storms translate to limnological storms, and ultimately impact phytoplankton community dynamics.

### 4.4 Sampling frequency, timing, and spatial coverage

The sampling frequency of long‐term monitoring programs typically ranges from weekly to monthly. Given that phytoplankton populations can double in time spans of one to a few days (Padisák, 2004), can such programs detect storm effects, and if so, under which conditions and at what time scales? If physical conditions within a lake rapidly return to prestorm conditions, community members that thrived prior to the storm may be able to weather the storm and demonstrate resistance, whereas rapid but seemingly temporary changes in lake environmental conditions can trigger a cascade of biological responses and interactions that produce long‐term impacts on phytoplankton dynamics (Kasprzak et al., 2017). For example, a comparison of routine manual fortnightly monitoring of phytoplankton to high‐frequency flow cytometry and fluorescence data revealed a cyanobacterial bloom that developed and then dissipated after a storm event between routine manual sampling dates (Pomati, Jokela, Simona, Veronesi, & Ibelings, 2011). The routine manual monitoring data, however, would have left the impression of community stability or stochastic changes in the absence of the high‐frequency sampling.

The frequency of data collection may also affect our perception of which factors are important drivers of phytoplankton dynamics on various timescales, potentially obscuring the real and relevant effects of storms (Havens et al., 2011; Thomas, Fontana, Reyes, Kehoe, & Pomati, 2018). As the chances of detecting a response to a storm event in the phytoplankton community decreases with increasing sampling interval (Bergkemper & Weisse, 2018), the occurrence and influence of abiotic (e.g., nutrient availability) and biotic (e.g., zooplankton grazing) fluctuations on phytoplankton dynamics likewise may not be captured using longer sampling intervals (Padisák, 1993). A sampling regime of once every 2 days is likely best suited to capture the influence of short‐term disturbances on phytoplankton dynamics (Edson & Jones, 1988; Padisák et al., 1988), but is highly labor‐intensive. Automated and semiautomated sampling techniques provide opportunities to improve resolution of data collected on phytoplankton community changes (Bergkemper & Weisse, 2018; Marcé et al., 2016), but, at best, can only provide information on major algal groups (Thomas et al., 2018) and cannot capture the taxonomic and functional resolution required to answer many ecological questions (but see Section 4.5 below). For example, automated sampling techniques can measure vertical phytoplankton distributions at high frequency, and thus provide highly resolved insights into how and to what extent storms affect vertical thermal structure and light conditions (Klug et al., 2012).

Another basic question, often reflected in the studies in our systematic review (Table 1), is how do we know if a change in community structure is a result of a storm or a “normal” seasonal trajectory on which a storm occurred (e.g., Paidere et al., 2007; Table S3)? Seasonal succession and reversions of phytoplankton communities have been at the core of the study of phytoplankton dynamics (Reynolds, 1980, 1988a, 1993). The plankton ecology group (PEG) model described the seasonal succession of phyto‐ and zooplankton as an annually repeated process of community assembly in lakes (de Senerpont Domis et al., 2013; Sommer et al., 1986, 2012), highlighting the relative importance of physical factors, grazing, parasites, nutrient limitation, fish predation, and food limitation. The PEG model provides a seasonal template that needs to be integrated into any consideration of how storm effects manifest, and highlights the importance of the timing of storms in relation to antecedent conditions. Similarly, for lakes on a long‐term changing environmental trajectory such as increasing eutrophication, a storm may appear to have a lasting impact but merely accelerated a “natural” progression (Bachmann et al., 1999; Clugston, 1963; Havens et al., 2001; James et al., 2008). Many of the studies in our systematic review observed that the trajectory of the communities and the duration of storm effects was highly variable and could be related to antecedent conditions (Perga et al., 2018) or a consequence of sampling frequency (James et al., 2008; Padisák, Tóth, & Rajczy, 1990).

Analyses of existing time series data can provide information on how data gaps (i.e., lower sampling frequency) impact pattern detection (Aguilera et al., 2016). Quantitative treatment of seasonal dynamics, such as continuous wavelet transforms to assess periodicity (Carey, Hanson, Lathrop, & St. Amand, 2016), hysteresis (Lloyd, Freer, Johnes, & Collins, 2016) and multitable multivariate analyses (Anneville et al., 2002) calculate deviations of observations from average seasonal trajectories to objectively assess if observed phytoplankton dynamics are a result of a storm, part of seasonal trajectories that happen to overlap with a storm, or perhaps a result of other factors (e.g., seasonality in top–down processes; Sommer et al., 2012).

Most sampling programs, including high‐frequency monitoring buoys and probes, are also limited to a single sampling point in a lake. Such a design is problematic in any system, but particularly in large lake systems due to their heterogeneous nature and internal physics (Liu et al., 2014; Rinke et al., 2009), and presents a continuing challenge because of logistical and funding constraints. In such systems, high‐frequency measurements of chlorophyll *a* at one sampling point might not measure, for example, phytoplankton growth responses after a storm but rather the wind‐induced horizontal or vertical shift of phytoplankton (Rinke et al., 2009). Remote sensing and autonomous underwater vehicles (AUV) are emerging technologies (see below) that will lead to better understanding of spatial patterning of phytoplankton.

### 4.5 Emerging technologies

Access to new technologies and their application across systems will be essential to advance limnology (Burford et al., 2019; Salmaso, Anneville, Straile, & Viaroli, 2018) and critical to increase mechanistic understanding of storm impacts on plankton communities. The increased use of high‐frequency monitoring systems over the last decade has provided high‐throughput environmental data to continue to fill knowledge gaps on the ecosystem impacts of short‐lived and episodic events (Jennings et al., 2012; Klug et al., 2012; Marcé et al., 2016). Moreover, development of in situ high‐frequency biological instruments such as scanning flow cytometry and fluoroprobes provide higher taxonomic‐specific biological information than standard chlorophyll *a* (Arnoldini, Heck, Blanco‐Fernández, & Hammes, 2013; Pomati et al., 2011; Thomas et al., 2018). Automated underwater imaging microscopes represent the most recent and promising tool to capture plankton dynamics in situ with high frequency (Reyes, Spaak, & Pomati, 2017—see http://www.aquascope.ch). Such instruments are also being used on both towed gear and AUV, which can be deployed during storms to collect fine‐scale spatial coverage of phytoplankton distributions (Scofield, Watkins, Weidel, Luckey, & Rudstam, 2017).

Complementary to these advances in biological sensing is the application of metagenomic tools to provide new perspectives in the evaluation of planktonic diversity and changes driven by environmental disturbances at different temporal and spatial scales, including extreme climatic events. Metabarcoding provides a flexible and affordable tool for rapid biodiversity assessment in aquatic ecosystems (Pesant et al., 2015), including microbial (Ruiz‐González, Niño‐García, Berggren, & del Giorgio, 2017; Tessler et al., 2017), eukaryotic (Khomich, Kauserud, Logares, Rasconi, & Andersen, 2017; Yi et al., 2017), and viral (Skvortsov et al., 2016) communities. For example, application of high‐throughput sequencing provided novel insights into the effects of upland terrestrial matter on the biodiversity of bacterial communities in headwater streams following rainstorm events, and quantitative polymerase chain reaction suggested alterations in the functional diversity of the bacterial community in nitrification and denitrification processes (Kan, 2018).

Earth observation using satellites offers the capability for frequent observations of water quality across multiple spatial and temporal scales, in ways that are not feasible with ground‐based water quality monitoring (Schaeffer et al., 2013). The European Space Agency, the National Aeronautics and Space Administration, and other national and international space agencies operate several satellite sensors developed for monitoring ocean water quality that may prove relevant to the monitoring of storm impacts on large lakes (e.g., MODIS‐Aqua, Envisat‐MERIS, and Sentinel‐3 OLCI). The sensors can retrieve optically active water quality constituents such as chlorophyll *a*, phycocyanin, total suspended matter, lake surface water temperature, colored dissolved organic matter, light attenuation, and Secchi depth (IOCCG, 2018). Products are now available to provide these data at sufficiently high spatiotemporal resolutions to understand storm impacts horizontally across a lake basin from 0.25 to 1.0 km pixel resolution (Neil, Spyrakos, Hunter, & Tyler, 2019), with observations available every 1–3 days to provide a synoptic picture of all (large) lakes across a region. Other satellite missions primarily designed for terrestrial applications, such as Landsat‐8 OLI and Sentinel‐2 MSI, can also retrieve chlorophyll *a* and total suspended matter at even higher spatial resolutions (10–60 m; Dörnhöfer & Oppelt, 2016). Earth observation of optical water quality, however, only observes the surface layer, so integration of remote sensing datasets with in situ water quality measures is essential, not only to validate surface measures, but also to get a full picture of the water column, particularly for observing impacts in deep lakes. Acquisition of highly resolved spatial data at timescales of 1–3 days across many lakes provides novel opportunities for comparative work. For example, satellite data could be used in a before‐after‐control‐impact design to assess impacts of lakes inside and outside of storm paths.

Widespread application of emerging technologies will pave the way to better understand how episodic extreme events, such as storms, can impact biological communities on short‐ and long‐term time scales in lakes.

### 4.6 Collaboration as a way forward

A unified effort by empiricists, theoreticians, modelers, limnologists, and watershed scientists will be required to develop and advance a synthetic framework of storm impacts on phytoplankton. Collaborative projects which make use of existing information and data, and advance new research, will play critical roles to advance our understanding of how storms impact lakes. With the advent of global networks (e.g., GLEON, NETLAKE, GLOBOLAKES, SITES AquaNet, LakeMIP, ISIMIP, Aquacosm, AEMON, MANTEL), collaborative approaches make coordinated research activities across sites and methods possible. Increased willingness and demand to share data openly (Hampton et al., 2015), accompanied by good data management (Boland, Karczewski, & Tatonetti, 2017; Wilkinson et al., 2016), can facilitate collaboration and enhance the transferability of findings. Instead of using single techniques, coordinated efforts and experimental design that cross boundaries among laboratory, field, and theoretical studies can pave the way to better understand the impacts of storms on phytoplankton communities and aquatic ecosystem dynamics (Burford et al., 2019). As storms are expected to continue to grow in impact due to climate change (IPCC, 2014; Seneviratne et al., 2012), the need for researchers to share data and models across disciplines, institutions, and nations is critical to advance our understanding of how phytoplankton communities will respond to EWEs.

## 5 CONCLUSIONS

Collectively, our framework suggests that the impact of storm events on lake conditions is not a simple consideration or a singular function of storm strength at a particular point in time and space. Enhanced understanding of storm impacts requires a watershed scale approach, considering the relationships among storm, lake, and watershed attributes. Furthermore, antecedent conditions and timescales of meteorological forcing, ecological response, and data collection are essential considerations. Many key questions remain: what attributes of storms, lakes and watershed are most impactful to the lake environment; what role does seasonal phenology play; how does the configuration of lakes and watersheds contribute to or ameliorate the impacts of storms; how do biological communities respond to changes in the lake environment; how persistent are the impacts of storms for lake ecosystems? The importance of ecological context in mediating storm impacts and the inherent heterogeneity in weather conditions globally challenge our ability to fully understand the impacts of storms on water quality, phytoplankton, and ecosystem processes. We can rise to this challenge. To do so, we need to continue existing and initiate new long‐term monitoring programs, couple such programs with high‐frequency sensors, integrate cross‐discipline approaches (e.g., remote sensing, weather forecasting, limnology, lake, and climate modelling), and share and analyze big and long‐term data.

## Supporting information

Supinfo

Supinfo

## Data Availability

No data were used in this manuscript.
