# Supplementary material for: Storm impacts on phytoplankton community dynamics in lakes
Source: Glob Chang Biol. 2020 Mar 5;26(5):2756–84. doi: 10.1111/gcb.15033 (PMC7216882; doi:10.1111/gcb.15033)
Supplement: Supplementary file 1 — Supinfo [file GCB-26-2756-s001.docx]

# Supplementary Materials

# Systematic Literature Review

On 22 June 2017, a Web of Science (University of South Florida (USF) Tampa library subscription) search was performed with the following keywords: phytoplank* and (storm* or wind* or hurricane* or monsoon* or cyclone* or disturbance*). The search yielded 4346 papers to screen. The metagear R package (v. 0.5; Lajeunesse, 2016) was used to generate abstract and title screening forms and to distribute screening effort. The titles and abstracts of these 4356 papers were divided randomly among 16 screeners and PDF screening forms had the following keywords highlighted: storm, wind, and rain. From the 4356 papers, 328 were coded as “possibly relevant” (composition of these papers: 243 observational, 22 experimental, and 60 models). Metagear was able to retrieve the full-text of 110 of the 328 relevant papers using the USF Tampa Library subscription; retrieval errors included 77 without DOI and 144 HTML errors. An additional 199 were retrieved manually. The remaining 19 papers were unretrievable, and were not included in our systematic review. Consequently, our systematic review included 94% (309) of the original 328 papers screened as possibly relevant.

Of the 309 papers with full-text available, effort was divided in a dual-coding design where 7 teams of 2 coders independently screened approximately 44 of the 309 papers for relevance. A paper was deemed “RELEVANT” for inclusion if it was about (1) storm effects on (2) phytoplankton in (3) lakes, reservoirs, or ponds. Papers not meeting these three criteria were coded as “NOT RELEVANT”. The form used to code the content of each paper can be found in Supplementary Form 1.

Papers deemed as RELEVANT also had their characteristics coded for systematic review summaries. All papers, regardless if deemed RELEVANT or NOT RELEVANT, were reviewed for a definition of “storm” and such definitions were recorded and summarized. Papers could also be coded as ANCILLARY if they reported tangential information relevant to our systematic review but were not the main effects reported in the original paper. For example, if a paper reported the potential for storm effects in the discussion only.

Overall, 118 of the 309 (38.2%) of all the coded papers used the word ‘storm’, but only 38 definitions were found in the 118 papers. The most frequently used words to define storm, including plural forms and stemmed-words, were: event (30), wind (28), storm (28), hurricane (16), speed (13), September (10), and rain (10).

Most of the 309 papers were deemed as NOT RELEVANT by both coders (Table S1). Only 19 papers were found to be RELEVANT by both coders. We had 28 papers with conflicting agreements between the two coders, and 10 papers with incomplete agreement (Table S1). Conflicting agreements were resolved if one coder screened a paper as RELEVANT and the second coded the same paper as ANCILLARY, which resulted in 17 of the 28 conflicting papers deemed RELEVANT. For the papers with incomplete agreement, papers were deemed RELEVANT if at least one coder coded the study as RELEVANT. Consequently, 2 of the 10 incomplete agreement papers were deemed RELEVANT, bringing the total number of papers deemed as RELEVANT to 38. We had a third coder screen the papers that had conflicting and incomplete agreements for relevance as an additional step. The third coder deemed that one of the two incomplete agreement papers deemed as RELEVANT was NOT RELEVANT, leaving a total of 37 papers deemed RELEVANT. As a final check, two coders assessed the 37 RELEVANT papers specifically for links of (1) storm effects on (2) physics/chemistry of lakes, reservoirs, or ponds with (3) evaluation of phytoplankton responses, and found only 25 of the 37 papers met these criteria (Table S2).

**Reference List for Supplementary Materials**

Aoki, T., Hayami, Y., Fujiwara, T., Mukai, H., & Tanaka, Y. (1996). Nutrient dynamics in the north basin of Lake Biwa: I. Changes in the vertical distribution of nutrients due to an internal surge induced by a strong typhoon. *Journal of Great Lakes Research, 22*, 331-340. https://doi.org/10.1016/S0380-1330(96)70959-0

Arfi, R. (2005). Seasonal ecological changes and water level variations in the Sélingué Reservoir (Mali, West Africa). *Physics and Chemistry of the Earth, 30,* 432-441. https://doi.org/10.1016/j.pce.2005.06.010

Baranyi, E., Tóth, L. G., & Homonnay, Z. G. (2011). The effect of variable turbulent intensities on the distribution of zooplankton in the shallow, large Lake Balaton (Hungary). *Knowledge and Management of Aquatic Ecosystems, 400,* 07. https://doi.org/10.1051/kmae/2011003

Barbiero, R. P., James, W. F., & Barko, J. W. (1999). The effects of disturbance events on phytoplankton community structure in a small temperate reservoir. *Freshwater Biology, 42,* 503-512. https://doi.org/10.1046/j.1365-2427.1999.00491.x

Bauer, A., & J. J. Waniek. (2013). Factors affecting chlorophyll a concentration in the central Beibu Gulf, South China Sea. *Marine Ecology Progress Series, 474,* 67-88. https://doi.org/10.3354/meps10075

Bleiker, W., & Schanz, F. (1997). Light climate as the key factor controlling the spring dynamics of phytoplankton in Lake Zürich. *Aquatic Sciences, 59,* 135–157. https://doi.org/10.1007/BF02523177

Blottière, L., Rossi, M., Madricardo, F., & Hulot, F. D. (2014). Modeling the role of wind and warming on *Microcystis aeruginosa* blooms in shallow lakes with different trophic status. *Theoretical Ecology, 7,* 35-52. https://doi.org/10.1007/s12080-013-0196-2

Bocaniov, S. A., Schiff, S. L., & Smith, R. E. H. (2012). Plankton metabolism and physical forcing in a productive embayment of a large oligotrophic lake: insights from stable oxygen isotopes. *Freshwater Biology, 57,* 481–496. https://doi.org/10.1111/j.1365-2427.2011.02715.x

Dix, N. G., Phlips, E. J., & Gleeson, R. A. (2008). Water quality changes in the Guana Tolomato Matanzas National Estuarine Research Reserve, Florida, associated with four tropical storms. *Journal of Coastal Research, 55,* 26-37. https://doi.org/10.2112/SI55-008.1

Edson, J. J., & Jones, R. C. (1988). Spatial, temporal, and storm runoff-related variations in phytoplankton community structure in a small, suburban reservoir. *Hydrobiologia, 169,* 353-362. https://doi.org/10.1007/BF00007558

de Faria, D. M., de Souza Cardoso, L., & da Matta Marques, D. (2017). Epiphyton dynamics during an induced succession in a large shallow lake: wind disturbance and zooplankton grazing act as main structuring forces. *Hydrobiologia, 788,* 267–280. https://doi.org/10.1007/s10750-016-3002-5

Frenette, J. J., Vincent, W. F., Legendre, L., & Nagata, T. (1996a). Size-dependent changes in phytoplankton C and N uptake in the dynamic mixed layer of Lake Biwa. *Freshwater Biology, 36,* 221-236. https://doi.org/10.1046/j.1365-2427.1996.00083.x

Frenette, J. J., Vincent, W. F., Legendre, L., & Nagata, T. (1996b). Size-dependent phytoplankton responses to atmospheric forcing in Lake Biwa. *Journal of Plankton Research, 18,* 371-391. https://doi.org/10.1093/plankt/18.3.371

Galat, D. L., Lider, E. L., Vigg, S., & Robertson, S. R. (1981). Limnology of a large, deep North American terminal lake, Pyramid Lake, Nevada, U.S.A. *Hydrobiologia, 82,* 281-317. https://doi.org/10.1007/BF00048722

Garneau, M. E., Posch, T., Hitz, G., Pomerleau, F., Pradalier, C., Siegwart, R., & Pernthaler, J. (2013). Short-term displacement of *Planktothrix rubescens* (cyanobacteria) in a pre-alpine lake observed using an autonomous sampling platform. *Limnology and Oceanography, 58,* 1892-1906. https://doi.org/10.4319/lo.2013.58.5.1892

Gierach, M. M., & Subrahmanyam, B. (2007). Satellite data analysis of the upper ocean response to Hurricanes Katrina and Rita (2005) in the Gulf of Mexico. *IEEE Geoscience and Remote Sensing Letters, 4,* 132-136. https://doi.org/[10.1109/LGRS.2006.887145](https://doi.org/10.1109/LGRS.2006.887145)

Hartshorn, N., Marimon, Z., Xuan, Z., Cormier, J., Chang, N. -B., & Wanielista, M. (2016). Complex interactions among nutrients, chlorophyll-a, and microcystins in three stormwater wet detention basins with floating treatment wetlands. *Chemosphere, 144,* 408-419. https://doi.org/10.1016/j.chemosphere.2015.08.023

Havens, K. E., Beaver, J. R., Casamatta, D. A., East, T. L., James, R. T. Mccormick, P., & ... Rodusky, A. J. (2011). Hurricane effects on the planktonic food web of a large subtropical lake. *Journal of Plankton Research, 33,* 1081-1094. <https://doi.org/10.1093/plankt/fbr002>

Holzmann, R. (1993). Seasonal fluctuations in the diversity and compositional stability of phytoplankton communities in small lakes in upper Bavaria. *Hydrobiologia, 249,* 101-109. https://doi.org/10.1007/BF00008846

Isles, P. D. F., Giles, C. D., Gearhart, T. A., Xu, Y. Y., Druschel, G. K., & Schroth, A. W. (2015). Dynamic internal drivers of a historically severe cyanobacteria bloom in Lake Champlain revealed through comprehensive monitoring. *Journal of Great Lakes Research, 41,* 818-829. https://doi.org/10.1016/j.jglr.2015.06.006

James, R. T., Chimney, M. J., Sharfstein, B., Engstrom, D. R., Schottler, S. P., East, T., & Jin, K. R. (2008). Hurricane effects on a shallow lake ecosystem, Lake Okeechobee, Florida (USA). *Fundamental and Applied Limnology, 172,* 273-287. https://doi.org/10.1127/1863-9135/2008/0172-0273

Jenkinson, A. F., & Collison, F. P. (1977). An initial climatology of gales over the North Sea. Synoptic Climatology Branch Memorandum No. 62, Meteorological Office, Bracknell.

Jennings, E., Jones, S. E., Arvola, L., Staehr, P. A., Gaiser, E., Jones, I. D., & ... De Eyto, E. (2012). Effects of weather-related episodic events in lakes: an analysis based on high-frequency data. *Freshwater Biology, 57,* 589-601. https://doi.org/10.1111/j.1365-2427.2011.02729.x

Jones, S. E., Chiu, C. Y., Kratz, T. K., Wu, J. T., Shade, A., & McMahon, K. D. (2008). Typhoons initiate predictable change in aquatic bacterial communities. *Limnology And Oceanography, 53,* 1319-1326. https://doi.org/10.4319/lo.2008.53.4.1319

Klarer, D. M., & Millie, D. F. (1994). Regulation of phytoplankton dynamics in a Laurentian Great Lakes estuary. *Hydrobiologia, 286,* 97-108. https://doi.org/10.1007/BF00008500

Kumar, A., Mishra, D. R., Equeenuddin, Sk. Md., Cho, H. J., & Rastogi, G. (2017). Differential impact of anniversary-severe cyclones on the water quality of a tropical coastal lagoon. *Estuaries and Coasts, 40,* 317–342. https://doi.org/10.1007/s12237-016-0172-3

Lajeunesse, M. J. (2016). Facilitating systematic reviews, data extraction and meta-analysis with the metagear package for R. *Methods in Ecology and Evolution,* *7,* 323-330. https://doi.org/10.1111/2041-210X.12472

Lebo, M. E., Reuter, J. E., Goldman, C. R., Rhodes, C. L., Vucinich, N., & Mosely, D. (1993). Spatial variations in nutrient and particulate matter concentrations in Pyramid Lake, Nevada, USA, during a dry period. *Canadian Journal of Fisheries and Aquatic Sciences, 50,* 1045-1054. https://doi.org/10.1139/f93-121

Lee, J. Y., Kim, J. K., Owen, J. S., Choi, Y., Shin, K., Jung, S., & Kim, B. (2013). Variation in carbon and nitrogen stable isotopes in POM and zooplankton in a deep reservoir and relationship to hydrological characteristics. *Journal of Freshwater Ecology, 28,* 47-62. https://doi.org/10.1080/02705060.2012.689999

Li, X., Huang, T. L., Ma, W. X., Sun, X., & Zhang, H. H. (2015). Effects of rainfall patterns on water quality in a stratified reservoir subject to eutrophication: Implications for management. *Science of the Total Environment, 521-522,* 27-36. https://doi.org/10.1016/j.scitotenv.2015.03.062

Lin, J., Tang, D., Alpers, W., & Wang, S. (2014). Response of dissolved oxygen and related marine ecological parameters to a tropical cyclone in the South China Sea. Advances in Space Research, 53, 1081-1091. https://doi.org/10.1016/j.asr.2014.01.005

Lohrenz, S. E., Fahnenstiel, G. L., Millie, D. F., Schofield, O. M. E., Johengen, T., & Bergmann, T. (2004). Spring phytoplankton photosynthesis, growth, and primary production and relationships to a recurrent coastal sediment plume and river inputs in southeastern Lake Michigan. *Journal of Geophysical Research*, *109*, C10S14. https://doi.org/10.1029/2004JC002383

Mackay, E. B., Jones, I. D., Thackeray, S. J., & Folkard, A. M. (2011). Spatial heterogeneity in a small, temperate lake during archetypal weak forcing conditions. *Fundamental and Applied Limnology, 179,* 27–40. https://doi.org/10.1127/1863-9135/2011/0179-0027

Nielsen, T. G., & Kiørboe, T. (1991). Effects of a storm event on the structure of the pelagic food web with special emphasis on planktonic ciliates. *Journal of Plankton Research, 13,* 35-51. https://doi.org/10.1093/plankt/13.1.35

Ollinger, D., & Bäuerle, E. (1998). The influence of weather conditions on the seasonal plankton development in a large and deep lake (L. Constance) - II. Water column stability derived from one-dimensional hydrodynamical models. Management of Lakes and Reservoirs during Global Climate Change (Eds., George et al.), pp. 57-70. Kluwer Academic Publishers.

Padisák, J., Tóth, L. G., & Rajczy, M. (1988). The role of storms in the summer succession of the phytoplankton community in a shallow lake (Lake Balaton, Hungary). *Journal of Plankton Research, 10,* 249-265. https://doi.org/10.1093/plankt/10.2.249

Padisák, J., Tóth, L. G., & Rajczy, M. (1990). Stir-up effect of wind on a more-or-less stratified shallow lake phytoplankton community, Lake Balaton, Hungary. *Hydrobiologia, 191,* 249-254. https://doi.org/10.1007/BF00026058

Paidere, J., Gruberts, D., Škute, A., & Druvietis, I. (2007). Impact of two different flood pulses on planktonic communities of the largest floodplain lakes of the Daugava River (Latvia). *Hydrobiologia, 592,* 303-314. https://doi.org/10.1007/s10750-007-0770-y

Painter, S. C., Finlay, M., Hemsley, V. S., & Martin, A. P. (2016). Seasonality, phytoplankton succession and the biogeochemical impacts of an autumn storm in the northeast Atlantic Ocean. *Progress in Oceanography, 142,* 72-104. https://doi.org/10.1016/j.pocean.2016.02.001

Pannard, A., Bormans, M., & Lagadeuc, Y. (2007). Short-term variability in physical forcing in temperate reservoirs: effects on phytoplankton dynamics and sedimentary fluxes. *Freshwater Biology, 52,* 12-27. https://doi.org/10.1111/j.1365-2427.2006.01667.x

Pannard, A., Bormans, M., & Lagadeuc, Y. (2008). Phytoplankton species turnover controlled by physical forcing at different time scales. *Canadian Journal of Fisheries and Aquatic Sciences 65,* 47-60. https://doi.org/10.1139/f07-149

Planas, D., & Paquet, S. (2016). Importance of climate change-physical forcing on the increase of cyanobacterial blooms in a small, stratified lake. *Journal of Limnology, 75,* [https://doi.org/](https://doi.org/10.1139/f07-149)10.4081/jlimnol.2016.1371

Rinke, K., Huber, A. M. R., Kempke, S., Eder, M., Wolf, T., Probst, W. N. & Rothhaupt, K. O. (2009). Lake-wide distributions of temperature, phytoplankton, zooplankton, and fish in the pelagic zone of a large lake. *Limnology And Oceanography, 54,* 1306-1322. https://doi.org/10.4319/lo.2009.54.4.1306

Robarts, R. D., Waiser, M. J., Hadas, O., Zohary, T., & MacIntyre, S. (1998). Relaxation of phosphorus limitation due to typhoon-induced mixing in two morphologically distinct basins of Lake Biwa, Japan. *Limnology and Oceanography, 43,* 1023-1036. https://doi.org/10.4319/lo.1998.43.6.1023

Rueda, F., Moreno-Ostos, E., & Cruz-Pizarro, L. (2007). Spatial and temporal scales of transport during the cooling phase of the ice-free period in a small high-mountain lake. *Aquatic Sciences, 69,* 115–128. https://doi.org/10.1007/s00027-006-0823-8

Schelske, C. L., Carrick, H. J., & Aldridge, F. J. (1995). Can wind-induced resuspension of meroplankton affect phytoplankton dynamics? *Journal of the North American Benthological Society, 14,* 616-630. https://doi.org/10.2307/1467545

Siswanto, E., Morimoto, A., & Kojima, S. (2009). Enhancement of phytoplankton primary productivity in the southern East China Sea following episodic typhoon passage. *Geophysical Research Letters, 36,* L11603. https://doi.org/10.1029/2009GL037883

Sommer, U., Padisák, J., Reynolds, C. S., & Juhász-Nagy, P. (1993). Hutchinson's heritage: the diversity-disturbance relationship in phytoplankton. *Hydrobiologia, 249,* 1-7. https://doi.org/10.1007/BF00008837

Son, S., Platt, T., Fuentes-Yaco, C., Bouman, H., Devred, E., Wu, Y., & Sathyendranath, S. (2007). Possible biogeochemical response to the passage of Hurricane Fabian observed by satellites. *Journal of Plankton Research, 29,* 687-697. https://doi.org/10.1093/plankt/fbm050

Takeuchi, T, & Yoshida, Y. (1999). Relationship between the blooms of *Alexandrium catanella* and the water quality or meteorological factors. *Nippon Suisan Gakkaishi, 65,* 826-832. http://pascal-francis.inist.fr/vibad/index.php?action=getRecordDetail&idt=1247381

Wang, H., Yin, C., Wang, W., & Lu, J. (2007). Algae trapping in the macrophyte-covered littoral zone and its importance for protecting alongshore drinking water supplies in Taihu Lake, P.R. China. *Fresenius Environmental Bulletin, 16*, 1087-1092.

White, P. S., & Pickett, S. T. A. (1985). Natural disturbance and patch dynamics: an introduction. The Ecology of Natural Disturbance and Patch Dynamics (Eds S. T. A. Pickett & P. S. White), pp. 3-13. Academic Press, Orlando, FL.

Wu, T. F., Qin, B. Q., Brookes, J. D., Shi, K., Zhu, G. W., Zhu, M. Y., & ... Wang, Z. (2015). The influence of changes in wind patterns on the areal extension of surface cyanobacterial blooms in a large shallow lake in China. *Science of the Total Environment, 518,* 24-30. https://doi.org/10.1016/j.scitotenv.2015.02.090

Wu, T. F., Qin, B. Q., Zhu, G. W., Luo, L. C., Ding, Y. Q., & Bian, G. Y. (2013). Dynamics of cyanobacterial bloom formation during short-term hydrodynamic fluctuation in a large shallow, eutrophic, and wind-exposed Lake Taihu, China. *Environmental Science and Pollution Research, 20,* 8546-8556. https://doi.org/10.1007/s11356-013-1812-9

Yang, Z., Zhang, M., Shi, X. L., Kong, F. X., Ma, R. H., & Yu, Y. (2016). Nutrient reduction magnifies the impact of extreme weather on cyanobacterial bloom formation in large shallow Lake Taihu (China). *Water Research, 103,* 302-310. https://doi.org/10.1016/j.watres.2016.07.047

Znachor, P., Zapomelova, E., Rehakova, K., Nedoma, J. & Simek, K. (2008). The effect of extreme rainfall on summer succession and vertical distribution of phytoplankton in a lacustrine part of a eutrophic reservoir. *Aquatic Sciences, 70,* 77-86. https://doi.org/10.1007/s00027-007-7033-x

**TABLE S1**  Summary of dual coding outcomes for study relevance among 309 papers to meet the criteria of (1) storm effects on (2) phytoplankton in (3) lakes, reservoirs, or ponds. Columns are screening outcomes from one coder, and rows are from the second coder. Papers marked as DID NOT CODE were not coded by at least one of the two coders.

|  | Not Relevant | Relevant | Did Not Code |
| --- | --- | --- | --- |
| Not Relevant | 252 | 7 | 3 |
| Relevant | 21 | 19 | 0 |
| Did Not Code | 5 | 2 | 0 |

**Table S2**  Definitions or descriptions of storms found in the systematic review. In some cases, more than one definition was provided.

| **Study** | **Definition** |
| --- | --- |
| Aoki, Hayami, Fujiwara, Mukai, & Tanaka (1996) | typhoon |
| Arfi (2005) | **(1)** "...stormy events can be particularly intense, featuring high winds and very heavy rains within a short time."  **(2)** "Storms successively feature a heavy drop in atmospheric pressure, high winds, cooler air temperatures and heavy rains the more often. Such events are generally short, lasting for one to three hours."  **(3)** monsoon |
| Baranyi, Tóth, & Homonnay (2011) | "The storm, when the daily mean wind speed of 1.8 m· s^-1^ increased to above 11 m· s^-1^" |
| Barbiero et al. (1999) | **(1)** "A disturbance event [storm] can be defined as 'any relatively discrete event in time that disrupts ecosystem, community or population structure and changes resources, substrate availability or the physical environment' (White & Pickett, 1985). We interpreted this to mean temporally well-defined events that results in the removal of a substantial percentage of the epilimnion (and hence the epilimnetic phytoplankton community), and produced a substantial increase in soluble nutrient levels (Sommer et al., 1993)."  **(2)** "...unambiguous, short-term increases in both epilimnetic flushing rate and nutrient concentration."  **(3)** "…flushing rates in excess of 0.09 d^-1^ …^"^ |
| Bauer & Waniek (2013) | "Wind events classified into tropical storm, typhoon, tropical depression, and strong wind events (> 10 m s^-1^)" |
| Bleiker & Schanz (1997) | "stormy cold wind (cube of wind velocity up to 0.5 10^3^ m^3^ s^-3^)" |
| Blottière, Rossi, Madricardo, & Hulot (2014) | "simulations with a 2-day storm event with wind speed at 8 m s^-1^" |
| Bocaniov, Schiff, & Smith (2012) | "strong and sustained wind events…The mean wind speed…approached 12 m s^-1^ (43.2 km h^-1^) with a maximum of 15 m s^-1^ (54 km h^-1^)." |
| de Faria, Cardoso, & da Matta Marques (2017) | "we considered the summer storm (day 20) [46.7 mm of precipitation; 29.5 m s^-1^ wind velocity] a disturbance" |
| Dix, Phlips, and Gleeson (2008) | hurricane |
| Edson & Jones (1988) | "The storms...supplied...water to the lake representing about 33 % and 50 % of the total lake volume…" |
| Frenette, Vincent, Legendre, & Nagata (1996a) | typhoon |
| Frenette et al. (1996b) | typhoon |
| Gierach & Subrahmanyam (2007) | hurricane |
| Hartshorn et al. (2016) | "total rainfall quantities greater than 0.25 inches [6.35 mm]" |
| Havens et al. (2011) | hurricane |
| James et al. (2008) | hurricane |
| Jennings et al. (2012) | **(1)** "abrupt changes in physical, chemical, and/or biological parameters that are distinct from previous background levels and are often driven by sudden changes in weather and in particular extremes in precipitation, wind or temperature."  **(2)** "With the exception of one of the four events at Yuan Yang, all meteorological conditions were greater than two standard deviations from the season mean"  **(3)** Wind speeds of episodic events ranged from 2.6 to 10.9 m s^-1^ and rainfall ranged from 38 to 443 mm day^-1^. |
| Klarer & Millie (1994) | rainfall |
| Kumar, Mishra, Equeenuddin, Cho, & Rastogi (2017) | cyclone |
| Lebo et al. (1993) | "Galat et al. (1981) reported intense dust storms at Pyramid Lake, especially in the spring, with prevailing westerly winds in excess of 20 m s^-1^." |
| Lee et al. (2013) | monsoon |
| Li et al. (2015) | **(1)** "heavy rainfall events"  **(2)** "Rainfall events represent disturbances to water bodies because they initiate changes in the environment and hydrological conditions"  **(3)** various precipitation amounts used to define heavy rainfall events: 70.5, 61.8, 101.4 134.1, and 102.4 mm day^-1^ |
| Lin, Tang, Alpers, & Wang (2014) | Typhoon |
| Lohrenz et al. (2004) | "Strong northerly winds and associated waves accompanying the advent of late winter storms" |
| Mackay, Jones, Thackeray, & Folkard (2011) | "intense, episodic forcing events i.e. storms" |
| Nielsen & Kiørboe (1991) | "strong winds of >15 m s^-1^" |
| Ollinger & Bäuerle (1988) | "a day with about three times the original wind speeds" |
| Paidere et al. (2007) | "heavy rainstorms" |
| Painter, Finlay, Hemsley, & Martin (2016) | **(1)** cyclones  **(2)** "We make use of the Jenkinson Gale Index to assess storm frequency and storm intensity in the northeast Atlantic (Jenkinson and Collison , 1977)...we classify gales based on the exceedance of certain threshold values of G, thus G > 30 indicates a gale, G > 40 a severe gale and G > 50 a very severe gale." |
| Robarts et al. (1998) | **(1)** "The typhoon, with wind speeds up to 20 m s^-1^…"  **(2)** "…the physical event is intense but of short duration (e.g. storms or typhoons)…" |
| Rueda, Moreno-Ostos, & Cruz-Pizarro (2007) | **(1)** "strong winds of episodic nature (storms)"  **(2)** "strong winds with speeds of 15 m s^-1^ and higher”  **(3)** "perturbations in atmospheric conditions characterized by large wind speeds or pulses of cold atmospheric masses (storms…)" |
| Siswanto, Morimoto, & Kojima (2009) | typhoon |
| Son et al. (2007) | hurricane |
| Takeuchi & Yoshida (1999) | "heavy rain or strong wind" |
| Wang, Yin, Wang, & Lu (2007) | hurricane |
| Wu et al. (2013) | tropical storm |
| Yang et al. (2016) | **(1)** "...we defined a daily average wind speed above 4 m/s as a strong wind event."  **(2)** "...we defined a daily average precipitation above 20 mm as a heavy rainfall event…" |

**Table S3**  Sampling frequency and the duration of the effects of storms on phytoplankton in each study. Sampling frequency occurred over multiple time scales in some studies. “Difficult” column indicates that observed changes in phytoplankton variables were difficult to discern if changes were due to storm effects or seasonal trajectories.

| Study | Duration of plankton effects | ≤ 1 week | 1-3 weeks | > 3 weeks | Difficult |
| --- | --- | --- | --- | --- | --- |
| Barbiero et al. (1999) | Generally < 1 week, 2-3 weeks after most severe events | x | x |  | x |
| Edson & Jones (1988) | Varied by taxonomic group and storm; either 5-8 days or remainder of summer (not consistent within group) | x |  | x | x |
| Frenette et al. (1996a) | Short-term (“days”), not discussed further | x |  |  |  |
| Frenette et al. (1996b) | Lag of 1-3 days, and short-term (“days”) | x |  |  |  |
| Garneau et al. (2013) | Within-day changes documented (4-day study) | x |  |  |  |
| Havens et al. (2011) | Over a year |  |  | x | x |
| Holzmann (1993) - Kautsee | Not differentiated from seasonal patterns; wind events discussed as catalysts of change |  | x |  | x |
| Holzmann (1993) - Thalersee | Not differentiated from seasonal patterns; wind events discussed as catalysts of change |  | x |  | x |
| Isles et al. (2015) | Not differentiated from seasonal patterns, discussed as catalyst | x |  |  | x |
| James et al. (2008) | Multiple years |  |  | x | x |
| Jennings et al. (2012) - Leane | Not addressed directly, reference made to < 8 days, 2-3 weeks, and also > year for physical changes related to biota | x | x | x |  |
| Jennings et al. (2012) - Slotssø | Same as above | x | x | x |  |
| Jones et al. (2008) | Only discussed with regard to bacteria | x |  |  |  |
| Li et al. (2015) | Duration depended on frequency and intensity of events and period of stratification (not specified, except an increase in frequent heavy rains depressed phytoplankton biomass “for a long time”; > 1 week implied) |  | x |  | x |
| Padisak et al. (1988) | Pre- and post-storm communities clearly distinct, duration/reversion not discussed | x |  |  | x |
| Padisak et al. (1990) | Within-day changes documented (24-hour study) | x |  |  | x |
| Paidere et al. (2007) - Dvietes | Not differentiated from seasonal patterns, discussed as catalyst |  | x |  | x |
| Padiere et al. (2007) - Skuku | Not differentiated from seasonal patterns, discussed as catalyst |  | x |  | x |
| Pannard et al. (2007, 2008) - La Cheze | Response within days, return not observed (> 3 weeks) |  |  | x | x |
| Pannard et al. (2007, 2008) - Rophemel | Same as above |  |  | x | x |
| Planas & Paquet (2016) | Within-day changes documented, duration not discussed | x |  |  |  |
| Rinke et al. (2009) | More focused on spatial than temporal, but plankton effects persisted longer than physical (differentiate at ~ 1 week) |  | x |  |  |
| Robarts et al. (1998) - Biwa North | Within week; focus on nutrient availability before/after | x |  |  |  |
| Robarts et al. (1998) - Biwa South | Same as above | x |  |  |  |
| Schelske et al. (1995) | Highly variable, evidence from diel to daily and weekly effects wind on phytoplankton biomass and activity | x | x |  |  |
| Wu et al. (2013) | Hourly, (dis)entrainment floating cyanobacteria in wind mixing | x |  |  |  |
| Wu et al. (2015) | Hourly (dis)entrainment cyanos to days (effect wind on size of surface bloom) and years (long term decrease in average wind speed promotes blooms) | x |  | x | x |
| Yang et al. (2016) | Nutrient run-off from storms promote cyano growth (days), wind resuspension benthic cyanos (hours), enhanced blooms (days to weeks) | x | x |  |  |
| Znachor et al. (2008) | Effects storms may last days - e.g. flushing but back to ‘normal’ within week to seasonal effects - changing phytoplankton succession | x |  | x | x |

**
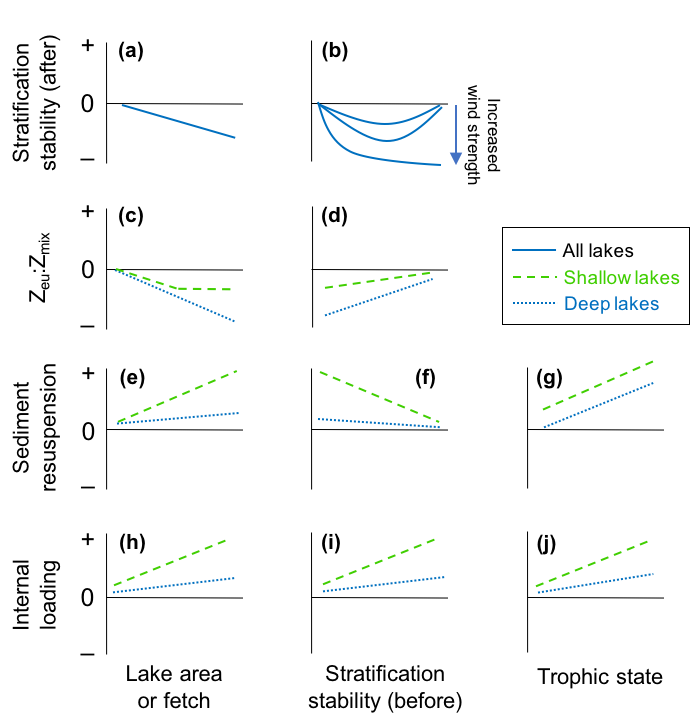
**

**FIGURE S1**  Expectations of how lake attributes and antecedent conditions (Figure 2a-c) will modify impacts of wind events on lake conditions (Figure 2d). Y-axis: horizontal zero line indicates no change before and after a wind event, a “+” indicates an increase in the metric, and a “–” indicates a decrease in the metric. Wind will increase mixing of the water column with subsequent effects on water column stability, light availability (Z_eu_:Z_mix_), sediment resuspension (inorganic and organic), and internal loading. The impacts may be expected to be similar across lakes of all depths, or may differ between shallow and deep lakes. In general, wind-induced mixing will reduce water column stability as lake size (area or fetch) increases (Panel a). The impacts of wind on light availability, as mediated by lake size, will be greater in deep lakes than shallow lakes because shallow lakes will be limited in their mixing depth Z_mix_ (i.e., lake bottom) compared to deep lakes (Panel c). Absence of any antecedent water column stability, or presence of very strong antecedent stability, will result in no change in water column stability after a wind event (Panel b). However, as wind strength increases we expect to see decreases in water column stability, with largest changes to occur when strong antecedent stability is mixed by strong winds (Panel b). Wind-induced mixing is expected to have a greater negative impact on light availability under conditions of weak antecedent stability (Panel d). Wind impacts on sediment resuspension should increase with lake size and trophic state, and decrease with increased antecedent stability, with stronger effects in shallow versus deep lakes (Panels e-g). Wind-induced impacts on internal nutrient loading will also increase with increased lake size, antecedent stability, and trophic state, with stronger effects in shallow lakes versus deeper lakes (Panels h-j). Internal nutrient loading will likely be sourced from the sediment or the sediment-water interface in shallow lakes and from hypolimnetic waters in deep lakes. In particular, strong antecedent stability in shallow, eutrophic lakes will likely promote release of phosphorus from sediments or facilitate nutrient build-up in hypolimnetic waters, priming lakes for high internal loading once mixed (Panel i).


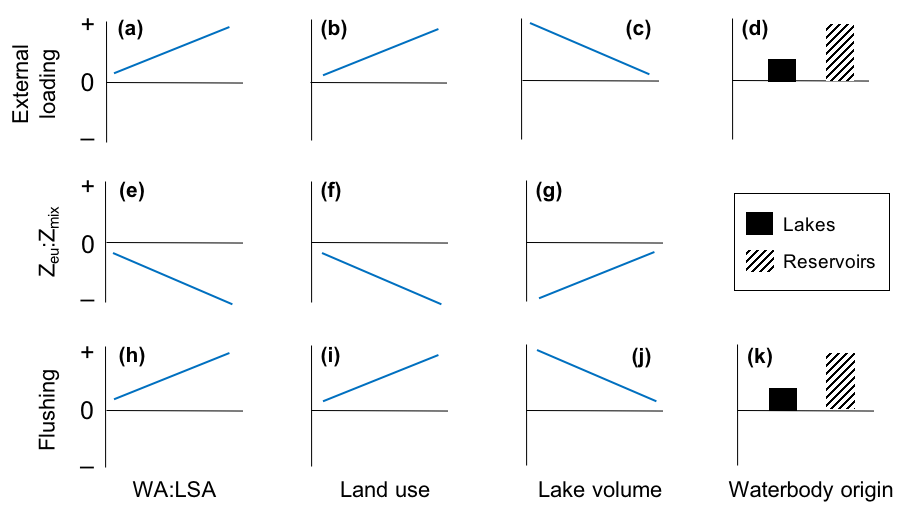


**FIGURE S2**  Expectations of how lake and watershed attributes (Figure 2a-c) will modify impacts of precipitation events on lake conditions (Figure 2d). Y-axis: horizontal zero line indicates no change before and after a wind event, a “+” indicates an increase in the impact, and a “–” indicates a decrease in the impact. precipitation is expected to impact lakes through delivery of external nutrient and sediment loads, decreased light availability due to external sediment loads, and increased flushing rates. Increased watershed area:lake surface area (WA:LSA) ratio and anthropogenic land use will positively impact external loading and negatively impact Z_eu_:Z_mix_ (i.e., through reduced light penetration) into lakes as a result of runoff from precipitation events. Conversely, increased lake volume is expected to dilute the effects of external loading in the lake and consequently reduce the negative impacts on Z_eu_:Z_mix_. WA:SLA and land use are expected to have a positive relationship with system flushing rates, whereas lake volume is expected to have a negative relationship with system flushing rate. For both external loading and system flushing rates, reservoirs are expected to be more impacted by precipitation events than natural lakes due to their “riverine”, dendritic nature and greater WA:LSA than lakes.
