# Supplementary material for: Storm impacts on phytoplankton community dynamics in lakes
Source: Glob Chang Biol. 2020 Mar 5;26(5):2756–84. doi: 10.1111/gcb.15033 (PMC7216882; doi:10.1111/gcb.15033)
Supplement: Supplementary file 2 — Supinfo [file GCB-26-2756-s002.pdf]

GEISHA

systematic review | v. 1.4 (4/27/18)

STUDY_ID [ ]

CODER LAST NAME [ ]

CONTENT OF PDF (needs to be coded for all of your assigned references)

Is this study about storm effects on phytoplankton in lakes/reservoirs/ponds? . . . . ☐

Checkmarking this box indicates that the *primary focus* was *storms*. This study can be observational, experimental, or a model.

If *NO*, is it relevant for other reasons? . . . . ☐

(e.g., effects of wind or rain but not necessarily extreme events, compared effects across a range of wind/rain intensities, discusses storm events, or is a review paper, editorial comment)

Note. If both boxes above are left empty, then this indicates that the study is not relevant, or does not contain relevant information, to our systematic review. For example, non-relevant publications include studies about marine systems, estuaries, or streams.

STORM INFORMATION

Was 'storm' defined? [NO = ☐ , if **YES** then copy/paste text description below]

Was the word 'storm' used in the publication? . . . . ☐

Use 'find' to search within text, but ignore uses in reference section.

In some studies, storms effects are not measured but only discussed. In what context were storms discussed in this study? copy/paste context and/or numerical thresholds

EXPERIMENTAL STUDY ATTRIBUTES

What was manipulated? . . . . [ ]

What was the response variable? . . . . [ ]

NOTE. If there are many, separate with commas.

Comments about study?

MODEL STUDY ATTRIBUTES

What was manipulated? . . . . [ ]

What was the response variable? . . . . [ ]

NOTE. If there are many, separate with commas.

Comments about study?

NOTES/COMMENTS

(optional) Was there anything of note about this study that was not covered by the coding form? Add notes/comments below…

STUDY MEASURES

MEASUREMENT CLASS

Note. If there are multiple sampling frequencies reported, then code the dominant or most abundant frequency. Fill ‘other’ below when the study reports a sampling frequency that is not available among the dropdown options.

WEATHER . . . . ☐

wind . . . . ☐ [sampling freq. = uncoded , other =]

rain . . . . ☐ [sampling freq. = uncoded , other =]

other . . . . [sampling freq. = uncoded , other =]

other . . . . [sampling freq. = uncoded , other =]

other . . . . [sampling freq. = uncoded , other =]

LAKE PHYSICS . . . . ☐

water temperature . . . . . ☐ [sampling freq. = uncoded , other =]

mixing/stratification . . . . ☐ [sampling freq. = uncoded , other =]

light . . . . ☐ [sampling freq. = uncoded , other =]

other . . . . [sampling freq. = uncoded , other =]

other . . . . [sampling freq. = uncoded , other =]

other . . . . [sampling freq. = uncoded , other =]

CHEMISTRY . . . . ☐

nutrient loading . . . . ☐ [sampling freq. = uncoded , other =]

(e.g., concentrations in nutrient measured in the water; covers both internal/external nutrient loading)

sediment loading . . . . ☐ [sampling freq. = uncoded , other =]

(e.g., sediment coming in from the watershed [via rivers], or sediment disturbed from lake bottom)

other . . . . [sampling freq. = uncoded , other =]

other . . . . [sampling freq. = uncoded , other =]

other . . . . [sampling freq. = uncoded , other =]

PHYTOPLANKTON . . . . ☐

diversity. . . . . ☐ [sampling freq. = uncoded , other =]

(e.g., richness, taxonomy, Shannon index)

composition . . . . ☐ [sampling freq. = uncoded , other =]

(e.g., abundance, dominance)

total abundance or biovolume/biomass . . . . ☐ [sampling freq. = uncoded , other =]

growth rate . . . . ☐ [sampling freq. = uncoded , other =]

chlorophyll . . . . ☐ [sampling freq. = uncoded , other =]

physiology . . . . ☐ [sampling freq. = uncoded , other =]

metabolism . . . . ☐ [sampling freq. = uncoded , other =]

(e.g., primary production, respiration using oxygen differences)

other . . . . [sampling freq. = uncoded , other =]

other . . . . [sampling freq. = uncoded , other =]

other . . . . [sampling freq. = uncoded , other =]

OTHER 1 . . . . [sampling freq. = uncoded , other =]

OTHER 2 . . . . [sampling freq. = uncoded , other =]

OTHER 3 . . . . [sampling freq. = uncoded , other =]

OTHER 4 . . . . [sampling freq. = uncoded , other =]

OTHER 5 . . . . [sampling freq. = uncoded , other =]

LAKE & WATERSHED ATTRIBUTES

uncoded **= type**
name . . . . [ ]

coordinates = [lat. = , long. =]
NOTE. Please use Google maps to determine *decimal coordinates* up to 3 decimals.
country = [ ]
study duration = [–]
surface area = [, units =]
max depth = [, units =]
mean depth = [, units =]
trophic status = uncoded **other =** [ ]
TP (total P) = [, units =]
mixing regime = uncoded **other =** [ ]
number of storm events identified/investigated = [ ]

uncoded **= type**
name . . . . [ ]

coordinates = [lat. = , long. =]
NOTE. Please use Google maps to determine *decimal coordinates* up to 3 decimals.
country = [ ]
study duration = [–]
surface area = [, units =]
max depth = [, units =]
mean depth = [, units =]
trophic status = uncoded **other =** [ ]
TP (total P) = [, units =]
mixing regime = uncoded **other =** [ ]
number of storm events identified/investigated = [ ]

uncoded **= type**
name . . . . [ ]

coordinates = [lat. = , long. =]
NOTE. Please use Google maps to determine *decimal coordinates* up to 3 decimals.
country = [ ]
study duration = [–]
surface area = [, units =]
max depth = [, units =]
mean depth = [, units =]
trophic status = uncoded **other =** [ ]
TP (total P) = [, units =]
mixing regime = uncoded **other =** [ ]
number of storm events identified/investigated = [ ]

uncoded **= type**
name . . . . [ ]

coordinates = [lat. = , long. =]
NOTE. Please use Google maps to determine *decimal coordinates* up to 3 decimals.
country = [ ]
study duration = [–]
surface area = [, units =]
max depth = [, units =]
mean depth = [, units =]
trophic status = uncoded **other =** [ ]
TP (total P) = [, units =]
mixing regime = uncoded **other =** [ ]
number of storm events identified/investigated = [ ]

uncoded **= type**
name . . . . [ ]

coordinates = [lat. = , long. =]
NOTE. Please use Google maps to determine *decimal coordinates* up to 3 decimals.
country = [ ]
study duration = [–]
surface area = [, units =]
max depth = [, units =]
mean depth = [, units =]
trophic status = uncoded **other =** [ ]
TP (total P) = [, units =]
mixing regime = uncoded **other =** [ ]
number of storm events identified/investigated = [ ]

uncoded **= type**
name . . . . [ ]

coordinates = [lat. = , long. =]
NOTE. Please use Google maps to determine *decimal coordinates* up to 3 decimals.
country = [ ]
study duration = [–]
surface area = [, units =]
max depth = [, units =]
mean depth = [, units =]
trophic status = uncoded **other =** [ ]
TP (total P) = [, units =]
mixing regime = uncoded **other =** [ ]
number of storm events identified/investigated = [ ]

uncoded **= type**
name . . . . [ ]

coordinates = [lat. = , long. =]
NOTE. Please use Google maps to determine *decimal coordinates* up to 3 decimals.
country = [ ]
study duration = [–]
surface area = [, units =]
max depth = [, units =]
mean depth = [, units =]
trophic status = uncoded **other =** [ ]
TP (total P) = [, units =]
mixing regime = uncoded **other =** [ ]
number of storm events identified/investigated = [ ]

uncoded **= type**
name . . . . [ ]

coordinates = [lat. = , long. =]
NOTE. Please use Google maps to determine *decimal coordinates* up to 3 decimals.
country = [ ]
study duration = [–]
surface area = [, units =]
max depth = [, units =]
mean depth = [, units =]
trophic status = uncoded **other =** [ ]
TP (total P) = [, units =]
mixing regime = uncoded **other =** [ ]
number of storm events identified/investigated = [ ]

uncoded **= type**
name . . . . [ ]

coordinates = [lat. = , long. =]
NOTE. Please use Google maps to determine *decimal coordinates* up to 3 decimals.
country = [ ]
study duration = [–]
surface area = [, units =]
max depth = [, units =]
mean depth = [, units =]
trophic status = uncoded **other =** [ ]
TP (total P) = [, units =]
mixing regime = uncoded **other =** [ ]
number of storm events identified/investigated = [ ]

uncoded **= type**
name . . . . [ ]

coordinates = [lat. = , long. =]
NOTE. Please use Google maps to determine *decimal coordinates* up to 3 decimals.
country = [ ]
study duration = [–]
surface area = [, units =]
max depth = [, units =]
mean depth = [, units =]
trophic status = uncoded **other =** [ ]
TP (total P) = [, units =]
mixing regime = uncoded **other =** [ ]
number of storm events identified/investigated = [ ]

uncoded **= type**
name . . . . [ ]

coordinates = [lat. = , long. =]
NOTE. Please use Google maps to determine *decimal coordinates* up to 3 decimals.
country = [ ]
study duration = [–]
surface area = [, units =]
max depth = [, units =]
mean depth = [, units =]
trophic status = uncoded **other =** [ ]
TP (total P) = [, units =]
mixing regime = uncoded **other =** [ ]
number of storm events identified/investigated = [ ]
